# Genome-wide identification and expression analysis of the plant specific *LIM* genes in *Gossypium arboreum* under phytohormone, salt and pathogen stress

**DOI:** 10.1038/s41598-021-87934-0

**Published:** 2021-04-28

**Authors:** K. P. Raghavendra, J. Das, R. Kumar, S. P. Gawande, H. B. Santosh, J. A. Sheeba, S. Kranthi, K. R. Kranthi, V. N. Waghmare

**Affiliations:** 1 Division of Crop Improvement, ICAR – Central Institute for Cotton Research (CICR), Nagpur, Maharashtra India; 2 Division of Crop Protection, ICAR – Central Institute for Cotton Research (CICR), Nagpur, Maharashtra India; 3 Division of Crop Production, ICAR – Central Institute for Cotton Research (CICR), Nagpur, Maharashtra India; 4 Technical Information Section, International Cotton Advisory Committee (ICAC), Washington, DC, USA

**Keywords:** Biotechnology, Plant sciences

## Abstract

Asiatic cotton (*Gossypium arboreum*) cultivated as ‘*desi* cotton’ in India, is renowned for its climate resilience and robustness against biotic and abiotic stresses. The genome of *G. arboreum* is therefore, considered as a valued reserve of information for discovering novel genes or gene functions for trait improvements in the present context of cotton cultivation world-wide. In the present study, we carried out genome-wide analysis of *LIM* gene family in desi cotton and identified twenty LIM domain proteins (GaLIMs) which include sixteen animals CRP-like GaLIMs and four plant specific GaLIMs with presence (GaDA1) or absence (GaDAR) of UIM (Ubiquitin Interacting Motifs). Among the sixteen CRP-like GaLIMs, eleven had two conventional LIM domains while, five had single LIM domain which was not reported in *LIM* gene family of the plant species studied, except in *Brassica rapa.* Phylogenetic analysis of these twenty GaLIM proteins in comparison with LIMs of Arabidopsis, chickpea and poplar categorized them into distinct αLIM1, βLIM1, γLIM2, δLIM2 groups in CRP-like LIMs, and GaDA1 and GaDAR in plant specific LIMs group. Domain analysis had revealed consensus [(C-X_2_-C-X_17_-H-X_2_-C)-X_2_-(C-X_2_-C-X_17_-C-X_2_-H)] and [(C-X_2_-C-X_17_-H-X_2_-C)-X_2_-(C-X_4_-C-X_15_-C-X_2_-H)] being conserved as first and/or second LIM domains of animal CRP-like GaLIMs, respectively. Interestingly, single LIM domain containing GaLIM15 was found to contain unique consensus with longer inter-zinc-motif spacer but shorter second zinc finger motif. All twenty *GaLIMs* showed variable spatio-temporal expression patterns and accordingly further categorized into distinct groups of αLIM1, βLIM1, γLIM2 δLIM2 and plant specific LIM (DA1/DAR). For the first time, response of *GaDA1/DAR* under the influence of biotic and abiotic stresses were studied in cotton, involving treatments with phytohormones (Jasmonic acid and Abscisic acid), salt (NaCl) and wilt causing pathogen (*Fusarium oxysporum*). Expressions patterns of *GaDA1/DAR* showed variable response and identified *GaDA2* as a probable candidate gene for stress tolerance in *G. arboreum.*

## Introduction

LIM proteins are cysteine-rich, homeodomain containing proteins named after **L**in11, **I**sl-1 and **M**ec-3 homeodomain proteins discovered in animals^1^. It contains consensus sequence [C-X_2_-C-X_16-23_-H-X_2_-C]-X_2_-[C-X_2_-C-X_16-21_C-X_2-3_-(C/D/H)] essentially known to have two zinc fingers linked together by a short two-amino acid spacer. LIM proteins with a novel cysteine-rich zinc-binding domain related to animal Cysteine Rich Proteins (CRPs) are described in mammals, amphibians, flies, worms and plants^2–7^.

The first report of LIM proteins in plant kingdom was documented in sunflower (*Helianthus annuus*), initially called as SF3 and subsequently, renamed as HaPLIM1^8,9^, which together with actin were known to participate in pollen development. Till now, the LIM domain containing proteins have been identified in several plant species including *Populus trichocarpa*, *Arabidopsis thaliana*, *Oryza sativa, Nicotiana tabacum*, *Glycine max, Gossypium hirsutum*, *Brassica rapa*, *Cicer arietinum*, *Pyrus bretschneideri* and *Solanum lycopersicum*^7,10–17^. Similar to animal CRPs, plant LIM proteins have also been localized in both cytosol and nucleus. Nuclear LIM domain proteins function as transcription factors involved in tissue specific gene regulation and cell fate determination. Whereas, cytoplasmic LIM domain proteins are known to actively participate in cytoskeleton organization through regulation of actin dynamics. Further, its dual functioning in congruence with site of cellular occurrence has also been well documented. In tobacco (*N. tabacum*), NtLIM2 protein exhibited twin role through actin-bundling and histone gene transcription^11^. Similarly, in cotton (*G. hirsutum*), GhWLIM1a and GhXLIM6 proteins showed altered fibre properties including fibre length, fineness and strength *via* modulating both F-actin dynamics and transcription of genes involved in phenyl-propanoid pathway^16,18^.

LIM domain proteins in plants have primarily been categorized into two subfamilies. The one similar to CRPs of animals contains LIM domain comprising of two zinc fingers linked together by a short two-amino acid spacer with some differences like long C-terminal and absence of glycine rich regions (GRR)^19^. While the second subfamily, known as plant specific LIM proteins, possess high molecular weight protein with single LIM domain, either presence (DA1) or absence (DAR) of UIMs (Ubiquitin Interacting Motifs) at N- terminal together and DUF3633 domain at C-terminal^13^. Based upon gene expression patterns, plant specific LIMs are further classified into two broad groups. Of which, pollen specific *LIM1* (*PLIM1*) and pollen-specific *LIM2* (*PLIM2*) exhibit preferential expressions during pollen development, while widely expressed *LIM1* and *LIM2* (*WLIM1* & *WLIM2*) shows predominant transcript abundances in the whole plant^20^. Plant specific DA1/DAR-LIM proteins are also reported to be key regulators of plant stress responses, including disease resistance and organ size^14,21,22^. Genome wide analysis of LIM protein gene family has been documented in several plants including *G. hirsutum*^23^, *A. thaliana*^19^, *P. trichocarpa*^7,24^, *O. sativa*^7^, *B. rapa*^12^, *P. bretschneideri*^17^, *S. lycopersicum*^15^, and *C. arietinum*^14^. Cotton (*Gossypium sp.*) fibre is basically an elongated single cell originating from ovular epidermis. Typically, the development phase of cotton fibre encompasses four overlapping stages of initiation, elongation, secondary cell-wall biogenesis and maturation^25,26^. The development of cotton fibre underlies a complex network of cellular signaling pathways involving the interplay of actin and microtubule dynamics^27^. Though, several research groups have attempted to unravel the gene regulatory network mediating cotton fibre development^26,28–30^, the vivid understanding of the same is still in its infancy.

LIM domain families of proteins are explored during recent times in upland cotton (*G. hirsutum*) to elucidate their roles during early fibre development and secondary cell-wall biogenesis^23^. Distinct roles of several LIM proteins (GhWLIM5, GhWLIM1a, GhXLIM6 and GhPLIM1) involved in regulating actin filament bundling, cellulose biogenesis during fibre and pollen development respectively, have been well characterized^16,31–33^. Among the *Gossypium* species, Asiatic cotton (*G. arboreum*) is one of the putative diploid progenitors (AA genome) of cultivated tetraploid cotton (*G. hirsutum*) which is known for its climate resilience against various biotic and abiotic stresses. Therefore, *G. arboreum,* as a study material is an apt choice for achieving sustainable cotton production in wake of widely perceived adverse impacts of climate change. Availability of *G. arboreum* complete genome sequence information provides a strong platform for next generation genomics assisted improvement of diploid cotton^34^. Systematic identification and characterization of biochemical, molecular and physiological factors contributing to the agronomical and economic traits of diploid progenitors will pave way for cotton improvement. *LIM* gene family of proteins therefore, harbors tremendous potential for their roles not only in fibre development of *G. hirsutum*, but also in stress physiology of wide genera of plants. This prompted us to carry out an in-depth study to explore *LIM* gene family in *G. arboreum* genome while understanding their potential roles in stress responses, both biotic and abiotic.

Therefore, in the present study, an *in silico* based comprehensive genome-wide analysis for *LIM* gene family members was carried out using *G. arboreum* genomic sequence. The identified putative candidates *LIM* gene members were analyzed for their genomic locations, gene structure, domain organization and ancestral phylogeny. Response of plant specific LIM members of *GaLIM*s were further studied for their responsiveness to challenges of biotic and abiotic stresses using real time PCR based gene expression profiling.

## Materials and methods

### Identification of the *GaLIM* genes in cotton

The protein database of cotton species *G. arboreum* (A_2_) ('SXY1' genome CGP-BGI_v2_a1)^34^, *G. raimondii* (D_5_) ('D5-4' genome NSF_v1)^35^ and *G. hirsutum* (AD_1_) ('TM-1' genome WHU_updated v1)^36^ were retrieved from CottonGen (https://www.cottongen.org**).** The hmm files of LIM domain (PF00412) and DUF3633 (PF12315) were extracted from Pfam database (http://pfam.xfam.org/) and was used as queries to scan the LIM protein sequences using HMMER software version 3.1b1^37^ with default parameters (E value < 0.001). Further, BLASTp and SMART tools (http://smart.embl-heidelberg.de/) were used to detect the conserved motifs of the putative LIM proteins. The gene structure was obtained after alignment of individual *LIMs* open reading frames with their respective gene on genome. The representation of the gene structure was performed by online Gene Structure Display Server (http://gsds.cbi.pku.edu.cn/).

### Chromosomal location and gene duplication

To trace chromosomal locations of *GaLIM* gene family members, relevant data were recovered from *G. arboreum* genome and subsequently mapped using MapChart software version 2.30^38^. Gene duplication events were analyzed as described in earlier studies^39,40^. Synonymous (Ks) and non-synonymous (Ka) substitution rates were determined using DnaSp software, followed by estimation of time of duplication events using the formula T = Ks/2λ, where clock-like rate (λ) denotes divergent time of 1.5 synonymous substitutions for every 108 years for cotton^41,42^.

### Sequence alignment, phylogeny, and protein analysis

The deduced LIM proteins of *G. arboreum* and that of other plant species (*P. trichocarpa, A. thaliana, C. arietinum*) were aligned. The alignment of sixty-one LIMs were used to generate phylogenetic tree using MEGA v.7.0 software^43^. Maximum Likelihood (ML) tree was constructed using JTT+G model. The ML tree was also generated for 37 DA1/DAR proteins comprising *G. arboreum*, *C. arietinum, B. rapa* and *G. max* using JTT+G model^43–45^. The bootstraps were performed for 1000 iterations and partial deletion was used for gap treatment. Number of amino acid residues, molecular weight, and pI value for each GaLIM protein was calculated using ProtParam tool (https://web.expasy.org/protparam). Subcellular localizations of GaLIM proteins were predicted using the online tool BaCelLo [**Ba**lanced Sub**cel**lular **Lo**calization **P**redictor]^46^.

### Plant material

The diploid cotton variety *G arboreum* cv. Roja, developed by ICAR-Central Institute for Cotton Research (ICAR-CICR), Nagpur was used in the present study. The seeds of *G. arboreum* cv. Roja were grown both in vitro on half strength of Murashige and Skoog’s (MS) plant tissue culture medium and *in-vivo* under field conditions at ICAR-CICR, Nagpur. For organ specific expression analysis, hypocotyl, cotyledonary leaves and root tissues were collected from 15 days old seedling raised on tissue culture MS medium^47^, whereas anthers and ovaries were collected from flowers at 0 days post-anthesis (DPA), ovules with fibres at 10 and 25 DPA respectively, representing fibre developmental stages collected from field grown plants. All the biological samples were collected in triplicate, immediately frozen in liquid nitrogen and stored at -80°C for gene expression studies.

### Stress treatments

#### Biotic stress

*G. arboreum* cv. Roja seedlings were inoculated with 10^6^ conidia/ml of *Fusarium oxysporum* f. sp. *vasinfectum* (NCBI accession No.MG372014.1), a pathogen causing cotton wilt and the infected plants were grown in glass house at ICAR-CICR, Nagpur. For inoculation, three weeks old cotton seedlings were immersed in the conidial suspension following root dip inoculation (RDI) method^48^. Samples were then collected from infected and mock-infected plants at 1 hour (h), 4h, 24h, 72h, and 96h, respectively. Upon collection, the samples were immediately frozen in liquid nitrogen and subsequently stored at -80°C until RNA isolation.

#### Hormonal treatments

Three weeks old glass house-grown cotton plants were treated through foliar spray with 100 μM of jasmonic acid (JA) and 100 μM of abscisic acid (ABA), two prominent plant defense hormones. The samples were collected from infected and mock-infected plants at 1h, 3h, 6h, 12h and 24h after the application of respective hormones.

#### Salt Stress treatment

Seven days old germinated seedlings were treated by drenching the plants with 100 ml of half strength Hoagland solution containing 200 mM NaCl. Leaf samples were collected and processed for RNA isolation after 1h, 3h, 6h, 12h and 24h of treatments. Plant seedlings treated with half strength Hoagland nutrient solution alone served as control.

### Gene expression analysis

#### Total RNA isolation and cDNA synthesis

Prior to RNA extraction, frozen plant samples were crushed with liquid nitrogen and ground to fine powder using pestle and mortar. For organ specific expression through RT-PCR, total RNA from leaf, root, hypocotyl, anther, ovary and fibre tissues from 10 DPA and 25 DPA were extracted using Spectrum Plant Total RNA kit (Sigma-Aldrich, USA) as per manufacturer's protocol. To quantify the transcript abundance of plant specific *LIM* genes (*GaDA1*/*DAR*), the total RNA each from abscisic acid (ABA), jasmonic acid (JA), sodium chloride (NaCl) treated and *F. oxysporum* challenged samples along with mock infected/ control samples were isolated. One microgram total RNA were quantified from each sample using Quantus Fluorometer (Promega, USA) and reverse transcribed into complementary DNAs (cDNAs) within a 20 μl reaction volume using GoScript Reverse Transcription System (Promega, USA) as directed by the manufacturer.

#### Quantitative Reverse Transcription PCR (qRT-PCR)

Twenty pairs of qPCR primers for *GaLIM* genes and normalizer *GhActin4* gene were designed with the aid of online Primer Quest tool (https://eu.idtdna.com) (Supplementary Table S1). The synthesized primers were later subjected to RT-PCR using cDNA samples for study gene expression studies. RT-PCR conditions encompassed 95 °C for 3 minutes, 95 °C for 40 seconds, 60 °C for 40 seconds, 72 °C for 40 seconds with 35 cycles followed by 72 °C for 7 minutes final extension. Similar conditions were simulated for qPCR experiments with an additional step for melt curve segment. Raw Ct value from three biological replicates in each treatment combinations were used to calculate fold change^49^. Significance of relative gene expression of each target gene was calculated using ANOVA.

## Results

### Genome-wide identification of *LIM* gene family in cotton

**Figure 1:**
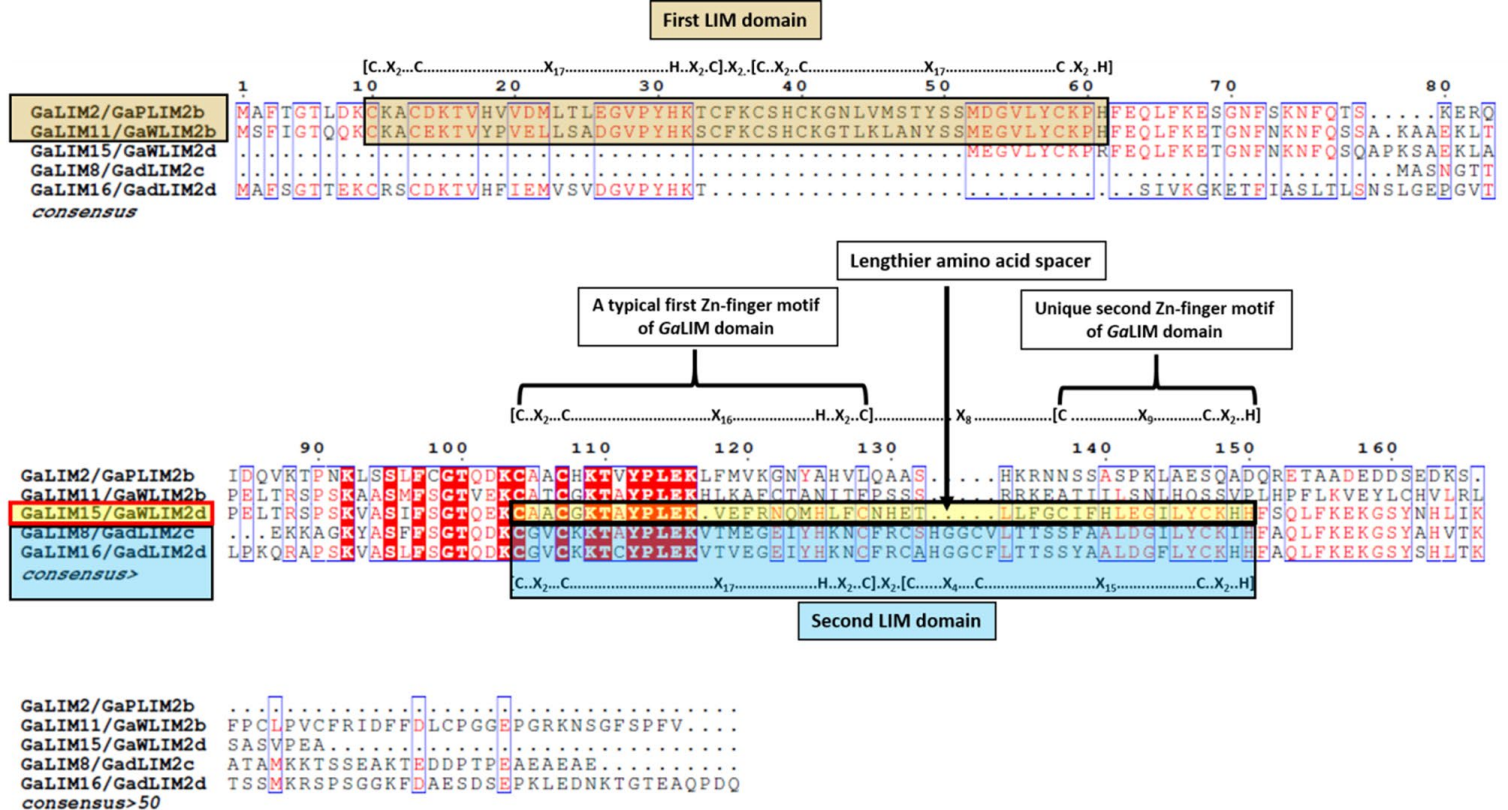
Multiple sequence alignment of single LIM domain GaLIM proteins.

The LIM domain Hidden Markov Model (PF00412) was used as a query to identify the candidate *LIM* genes in the *G. arboreum, G. raimondii* and *G. hirsutum* genome database through HMMER software tool. After removing redundant sequences, a total of 20, 18 and 35 unique *LIM* gene sequences containing at least a single LIM domain, were retrieved from *G. arboreum, G. raimondii* and *G. hirsutum* databases respectively (Table 1). Based upon their respective genome locations, *LIM* genes from *G. arboreum* were subsequently named as *GaLIM1* to *GaLIM20* (Table 1). Pair-wise amino acid analysis of these 20 GaLIM proteins showed 10.91 to 92.02% similarity (Supplementary Table S2). Four GaLIM proteins depicted identity of more than 80 percent among themselves, indicating their close relatedness. GaLIM14 and GaLIM20 were observed to be more closely related with 92.02% similarity in protein sequence. BaCelLo prediction revealed nuclear localization of eighteen GaLIMs except GaLIM4/PLIM2c and GaLIM18/GaDAR1 which were predicted to be secretory and chloroplast localized proteins, respectively (Table 1). SMART analysis of the identified protein sequences grouped the LIM proteins of *G. arboreum* into two subfamilies (Supplementary Fig. S1 & S2). The first subfamily was comprised of sixteen GaLIM proteins similar to animal CRPs and pair-wise amino acid analysis of those proteins showed a similarity index ranging between 31.94 to 92.02% (Supplementary Table S3). Out of these sixteen GaLIMs, eleven proteins had features of two LIM domains (2LIM) with amino acids ranging from 188 to 227 (21.06 to 25.10 kDa) (Table1 & Supplementary Fig. S3). Both the LIM domains shared consensus [(C-X_2_-C-X_17_-H-X2-C)-X_2_-(C-X_2_-C-X_17_-C-X_2_-H)] and [(C-X_2_-C-X_17_-H-X_2_-C)-X_2_-(C-X_4_-C-X_15_-C-X_2_-H)] representing first and second LIM domains, respectively (Supplementary Fig. S4). Notably, in addition to this, five proteins were predicted with single LIM domain (1LIM) with size ranging between 118 (12.8 kDa) and 202 (22.45 kDa) amino acids (Table 1). Among those 5 1LIM proteins, GaLIM2 and GaLIM11 shared a common LIM domain sequence consensus identical to that of first LIM domain of 2LIM, whereas GaLIM8 and GaLIM16 contained consensus related to second LIM domain of 2LIM (Fig. 1). GaLIM15 had a unique LIM domain consensus of [(C-X_2_-C-X_16_-H-X_2_-C)-X_8_-(C-X_9_-C-X_2_-H)]. Distinctively, GaLIM15 had relatively shorter second zinc finger motif due to a missing cysteine residue at first position (Fig. 1). In addition, it was also featured with lengthier spacer of eight amino acids between two Zn-finger motifs, unlike commonly observed 2-3 amino acid spacers in other LIM domains. The identification of single *LIM* genes related to animal CRPs, are rather unique to *G. arboreum* and interestingly, those are the first of their kinds amongst all *LIMs* reported across plant kingdom unless otherwise mentioned. Second subfamily of GaLIM was categorized as plant-specific LIM family of proteins consisting of single LIM domain along with DUF3633 at C-terminal and presence (DA1) or absence (DAR) of UIMs (Ubiquitin Interaction Motifs) at N- terminal (Supplementary Fig. S5 & S6). Four members of GaDA1/DAR proteins were identified under this subfamily with three belonging to DA1 and one to DAR (Supplementary Fig. S2). Predicted size of the GaDA1/DAR ranged from 472 to 633 amino acids (54.11 to 72.70 kDa) which also rate those as higher molecular weight proteins as compared to animal CRPs. Isoelectric point (pI) for GaLIM protein family ranged from 5.17 to 9.15 (Table 1).

**Table 1:** Details of *LIM* family genes identified in *G. arboreum* and their phylogenetic relationship in *G.raimondii* and *G .hirsutum*.

Gene ID	LIM Name	Chromosome location	Length of AAs	LIM domain Start–end (aa)	Mol.Wt. (kD)	pI	Number of Introns	Subcellular Localization	*Orthologous gene ID(Gene name) in*	
									*G raimondii*	*G hirsutum*^*b*^
Cotton_A_09995	*GaLIM1/GaδLIM2a*	3	217	9–61, 104–156	23.81	6.55	2	Nucleus	D5.v1.pred00011149 (GrLIM1/)	Ghi A08G11351/Ghi D08G10666(GhLIM1 A_t_/D_t_ )
Cotton_A_29508	*GaLIM2/ GaPLIM2b*	3	165	9–61	18.41	8.04	4	Nucleus	D5.v1.pred00010592 (GrLIM2)	Ghi A08G08411/Ghi D08G08016(GhLIM2 A_t_/D_t_ )
Cotton_A_23003	*GaLIM3 / GaPLIM2a*	3	188	9–61, 96–148	21.06	7.55	3	Nucleus	D5.v1.pred00009243 (GrLIM3)	Ghi A08G01906/Ghi D08G01886(GhLIM3 A_t_/D_t_ )
Cotton_A_21476	*GaLIM4 / GaPLIM2c*	4	209	9–61, 104–156	23.31	7.12	4	Secretory	D5.v1.pred00007880 (GrLIM4)	Ghi A11G04726/Ghi D11G05071(GhLIM4 A_t_/D_t_ )
Cotton_A_26589	*GaLIM5 / GaβLIM1a*	6	189	10–62, 108–160	21.24	9.03	4	Nucleus	D5.v1.pre 00,015,355 (GrLIM5)	Ghi A12G15831/Ghi D12G16341(GhLIM5 A_t_/D_t_ )
Cotton_A_33035	*GaLIM6 / GaDA1*	7	472	UIM:39–58, 70–89 LIM:110–162 DUF3633:260–467	54.11	5.64	10	Nucleus		Ghi A01G05801(GhLIM6 A_t_ )
Cotton_A_14459	*GaLIM7 / GaδLIM2b*	7	227	9–61, 104–156	25.10	5.42	4	Nucleus	D5.v1.pred00015920 (GrLIM7)	Ghi A12G13276/Ghi D12G13821(GhLIM7 A_t_/D_t_ )
Cotton_A_11634	*GaLIM8 / GaδLIM2c*	7	118	24–76	12.80	7.6	1	Nucleus	D5.v1.pred00040452 (GrLIM8)	Ghi A03G01221/Ghi D03G09036(GhLIM8 A_t_/D_t_ )
Cotton_A_13720	*GaLIM9 / GaDA2*	8	633	UIM199-218, 230–249; LIM:272–322; DUF3633:420–628	72.70	7.17	14	Nucleus	D5.v1.pred00002142 (GrLIM9)	GhiA05G12096/Ghi D05G07266(GhLIM9 A_t_/D_t_ )
Cotton_A_24851	*GaLIM10 / GaDA3*	A8	558	UIM:40–59, 142–161; LIM:182–234; DUF3633:332–553	63.16	5.17	10	Nucleus	D5.v1.pred00017888 (GrLIM10)	
Cotton_A_22981	*GaLIM11 / GaWLIM2b*	8	202	9–61	22.45	9.09	4	Nucleus	D5.v1.pred 00,032,771 (GrLIM11)	Ghi A06G10496/Ghi D06G10131(GhLIM11 A_t_/D_t_ )
Cotton_A_14838	*GaLIM12 / GaWLIM1a*	8	190	9–61, 109–161	21.05	9.03	4	Nucleus	D5.v1.pred 00,030,780 (GrLIM12)	Ghi A06G01216/Ghi D06G01081(GhLIM12 A_t_/D_t_ )
Cotton_A_21729	*GaLIM13 / GaFLIM1a*	8	196	10–62, 108–160	22.14	8.65	4	Nucleus	D5.v1.pred 00,025,044 (GrLIM13)	Ghi A03G11906/Ghi D02G11391(GhLIM13 A_t_/D_t_ )
Cotton_A_13549	*GaLIM14 / GaWLIM2c*	10	189	9–61, 106–158	20.82	9.15	3	Nucleus	D5.v1.pred 00,012,209 (GrLIM14)	GhiA10G14046/GhiD10G12816(GhLIM14 A_t_/D_t_ )
Cotton_A_32215	*GaLIM15 / GaWLIM2d*	10	126	57–103	14.33	8.74	4	Nucleus		
Cotton_A_02312	*GaLIM16 / GaδLIM2d*	11	182	78–130	19.90	8.52	3	Nucleus	D5.v1.pred 00,008,559 (GrLIM16)	Ghi D11G01066/Ghi A11G01191(GhLIM16 A_t_/D_t_ )
Cotton_A_06392	*GaLIM17 / GaFLIM1b*	12	208	10–62, 108–160	23.20	9.11	4	Nucleus	D5.v1.pred 00,036,622 (GrLIM17)	Ghi A04G06521/Ghi D04G09176(GhLIM17 A_t_/D_t_ )
Cotton_A_25109	*GaLIM18 / GaDAR*	13	517	LIM-152–204 DUF3633-302–509	57.84	8.26	11	Chloroplast	D5.v1.pred 00,012,313 (GrLIM18)	Ghi A10G13846/Ghi D10G13461(GhLIM18 A_t_/D_t_ )
Cotton_A_31990	*GaLIM19 / GaPLIM2d*	13	208	9–61, 102–154	23.25	6.81	4	Nucleus	D5.v1.pred 00,017,288 (GrLIM19)	Ghi A12G06686/Ghi D12G07496(GhLIM19 A_t_/D_t_ )
Cotton_A_00967	*GaLIM20 / GaWLIM2a*	13	189	9–61, 106–159	20.74	9.08	4	Nucleus	D5.v1.pred 00,018,816 (GrLIM20)	Ghi A13G00891/Ghi D13G00866(GhLIM20 A_t_/D_t_ )

In case of *G. raimondii* three plant specific *LIM* genes (two *GrDA1* and one *GrDAR*) were identified, while five were traced in *G. hirsutum* (three *GhDA1* and two *GhDAR*). Concurrently, there were 15 and 30 animal-CRP like *LIM* genes observed in *G. raimondii* and *G. hirsutum*, respectively*,* of which three and five LIM members from *G. raimondii* and *G. hirsutum* respectively, contained 1LIM domain each (Table 1; Supplementary Table S5 & S6).

### Genomic localization and gene structural analysis

**Figure 2:**
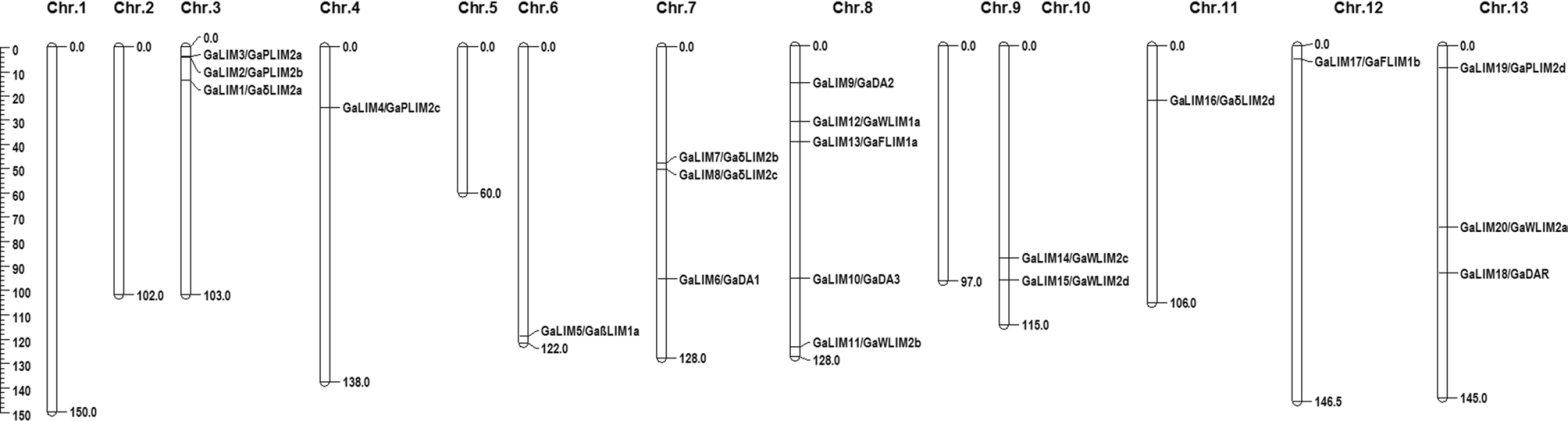
Chromosomal localization of *GaLIM* genes. (MapChart software version 2.30; https://www.wur.nl/en/show/Mapchart.htm).

MapChart analysis revealed that the identified 20 *GaLIM*s were unevenly distributed among the chromosomes of *G. arboreum* covering 9 chromosomes out of total 13 (Chr.1 to Chr.13). A maximum number of five *GaLIM*s were found to be localized on Chr.8, while three each were observed on Chr.3, Chr.7 and Chr.13 and two on Chr.10 respectively. Chromosomes 4, 6, 11 and 12 contained one *GaLIM* each, whereas chromosomes 1, 2, 5 and 9 were devoid of any *GaLIM*. Plant specific *GaLIM* (*DA1/DAR*) family members showed their localization on Chr.7, Chr.8 and Chr.13 (Table 1 & Fig. 2). Structural analysis of genomic regions with corresponding CDS revealed the organization of exon and intron in the *GaLIM* genes. Number of introns in each of the 20 *GaLIM* gene sequences ranged from 1 to 14 (Table 1). In the first subfamily of *LIM* genes similar to animal CRPs, 8 out of 16 genes showed 4 introns. Interestingly, only one intron was noticed on single LIM domain containing *GaLIM8* gene. Among second sub-gene family belonging to plant-specific group, maximum 14 introns were observed on genomic sequence of *GaLIM9* (*GaDA2*) and 10 introns each on *GaLIM*6 (*GaDA1*) and *GaLIM10* (*GaDA3*). Only one member of *GaDAR1* (*GaLIM18*) showed presence of 11 introns (Table 1 & Supplementary Fig. S7).

### Phylogenetic analysis and nomenclature of *GaLIM* genes

**Figure 3:**
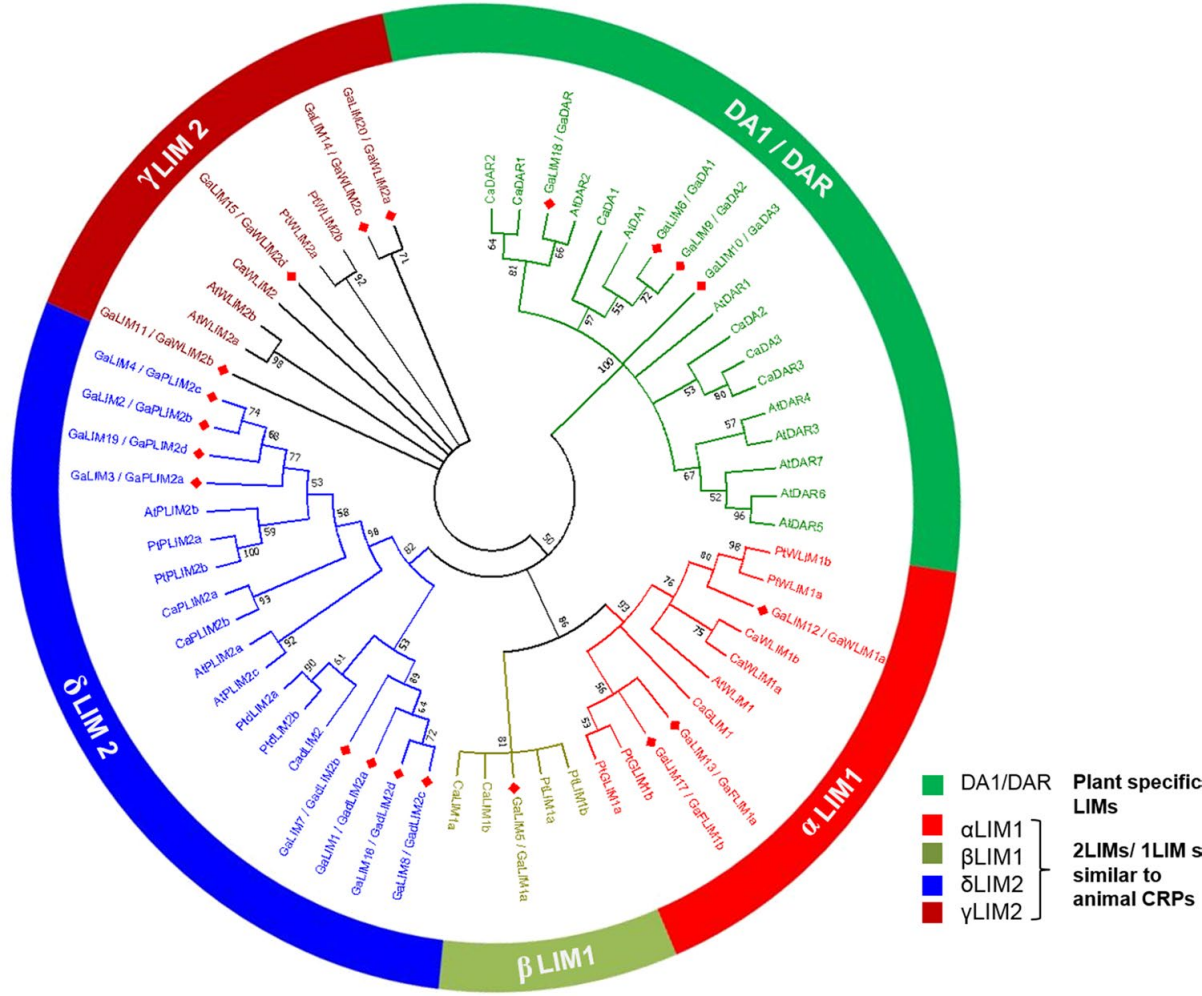
Maximum likelihood tree representing evolutionary relationship of the identified GaLIMs compared with LIMs from Arabidopsis, Poplar and Chickpea (MEGA v.7.0 software; https://www.megasoftware.net/).

To understand the phylogenetic relationships among *GaLIMs*, the amino acid sequences of LIM proteins from *A. thaliana*, *P. trichocarpa, C. arietinum* were retrieved from public database and published literatures (Supplementary data, SD1**).** A phylogenetic tree was constructed utilizing 20 GaLIMs, 14 AtLIMs (Arabidopsis), 12 PtLIMs (poplar) and 15 *Ca*LIMs (chickpea), using MEGA 7.0 software. The unrooted phylogenetic tree clearly formed two distinct groups with 2LIM/1LIM similar to animal CRPs and plant-specific DA1/DAR (Fig. 3). Four GaLIMs were found to be grouped with plant-specific DA1/DAR LIMs from Arabidopsis and chickpea and hence, renamed as GaDA1 and GaDAR. Phylogenetic analysis had revealed that *G. arboreum* DA1 and DAR LIM formed two distinct subgroups, where GaDA1, GaDA2 and GaDA3 amino acid sequences shared 55.84, 52.67 and 59.35 percent identities with respect to GaDAR and 68.35 to 85.38 percent among themselves (Supplementary Table S4). The 2LIM proteins from *G. arboreum* (16 LIMs), Arabidopsis (6 LIMs)*,* poplar (12 LIMs) and chickpea (9 LIMs) resulted in the segregation of 16 GaLIMs into four groups namely αLIM1, βLIM1, γLIM2 and δLIM2 as supported by high bootstrap values (Fig. 3). Subsequently, the members of GaLIMs clustered within those four groups were renamed according to their tissue-specific expression patterns by adopting the nomenclature described by Arnaud et al.^7^.

### Gene duplication

Paralogous gene pairs *GaLIM*s were analyzed to gain needed insights into the nature of gene duplication occurred in the LIM gene family in *G. arboreum.* Based on the gene and protein sequence similarities, 18 pairs of LIM genes were identified as putative paralogous among the *GaLIM* family. Genomic localization of gene pairs on different chromosomes for more than 15 gene pairs clearly substantiated the segmental duplications as the major means of amplification of *LIM* gene family in *G. arboreum* except for the 3 gene pairs showed dispersed gene duplications. Calculation of approximate time of gene duplication in the evolution of *GaLIM*s varied between 1.81 to 28.57 million years ago (mya) with an average of 11.12 mya (Supplementary Table S7).

### Organ-specific gene expression and confirmation of phylogeny

**Figure 4:**
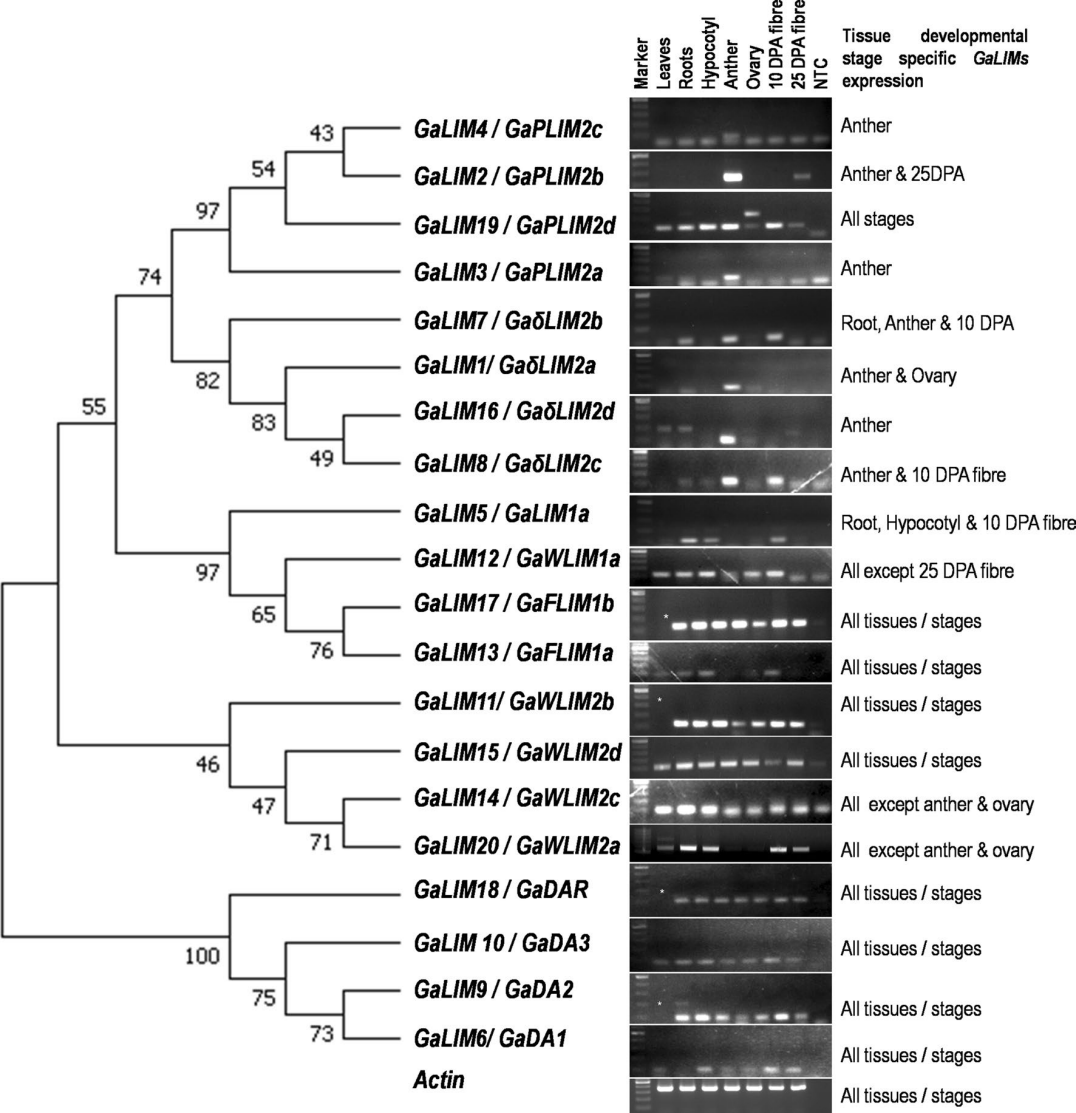
RT-PCR showing organ-specific expression of *LIM* genes in *G. arboreum.* (*denotes blank lane; Full-length gels are presented in Supplementary Figure S9).

Tissue and/or developmental stage specific gene expressions of 20 *GaLIM*s were confirmed through semi-quantitative PCR utilizing the cDNA as template from the vegetative tissues (root, leaf and hypocotyl), floral tissues (anther and ovary) and fibres in the developmental stage (10 DPA and 25DPA) of *G. arboreum* cv. Roja (Fig. 4). As mentioned earlier, these 20 *GaLIM*s could be grouped into five major groups such as *αLIM1*, *βLIM1*, *γLIM2*, * δLIM2* and plant specific *LIM* (*DA1/DAR*) groups based upon their gene expression patterns which also confirms the deduced phylogenetic relationships as well.

#### αLIM1 group

αLIM1 group comprises of three members namely GaWLIM1a, GaFLIM1a and GaFLIM1b. The pair-wise amino acid sequence similarities among them ranged from 76.06 to 79.59 percent and had shown their transcript abundance in all tissues of cotton under study. *GaFLIM1a* and *GaWLIM1a* manifested similar pattern of expression in all tissues/stages, preferentially during primary cell wall synthesis of cotton fibre (10 DPA) of cotton (Fig. 4). Notably, *GaFLIM1b* exhibited elevated expressions in all stages and tissues under study including both early (10 DPA) and advanced (25 DPA) stages of cell wall biosynthesis of cotton fibre.

#### βLIM1 group

*GaβLIM1* is the lone member observed under this group which portrayed similar pattern of gene expression as that of GaαLIM1group members, with preferential expressions being recorded at root, hypocotyl and cotton fibre at 10 DPA (Fig. 4).

#### γLIM2 group

It featured the members from WLIM2 subgroups with four duplicated members of GaWLIM2. Among those, *GaWLIM2a* and *GaWLIM2c* showed constitutively high expression in all the tissues and stages of cotton under study and shared 92.02 percent pair-wise identity at amino acids level. In contrast, gene expressions for *GaWLIM2b* and *GaWLIM2d* were observed in all the tissues, except reproductive parts (anther and ovary).

#### δLIM2 group

The δLIM2 group comprises of three monophyletic subgroups with members from monocot PLIM2, eudicot PLIM2 and asterids δLIM2. Upon phylogenetic comparison of GaLIMs with members of δLIM2 groups from *Arabidopsis*, chickpea and poplar, a subtotal of eight GaLIMs have been assigned to δLIM2 group. Those GaLIMs were further separated into two monophyletic subgroups namely PLIM2 and δLIM2 (PLIM2-like) based on expression patterns in the pollen development. Both PLIM2 and δLIM2 were comprised of four members each, where three out of four *GaPLIM2* (*GaPLIM2a*, *2b* and *2c*) showed preferential expressions in anther tissues. However, *GaPLIM2d* was found to be expressed in other tissues along with anther. Similarly, in δLIM2 (PLIM2-like) subgroup, all the members showed high expressions during anther development and in immature cotton fibre at 10 DPA stages, except *GaδLIM2d* which recorded its expression exclusively in anther development stage (Fig. 4). In addition, pair-wise amino acid sequence analysis of those four GaPLIM2 proteins showed similarity range of 76.22 to 82.61 percent indicating their close relatedness and probable duplication during evolution (Supplementary Table S2).

### Expression patterns of plant-specific LIM (DA1/DAR) group

All the members of plant-specific *GaLIM* (*DA1*/*DAR*) showed constitutive expressions in all the tissues and stages, with an exception of *GaDA1* having no detectable expression in root tissues. Notably, *GaDA2* showed superior expression level as compared to that of *GaDA1* or *GaDA3* or *GaDAR1* (Fig. 4).

### Expression analysis of plant-specific *LIM* genes in response to stresses

#### Hormonal treatment

**Figure 5:**
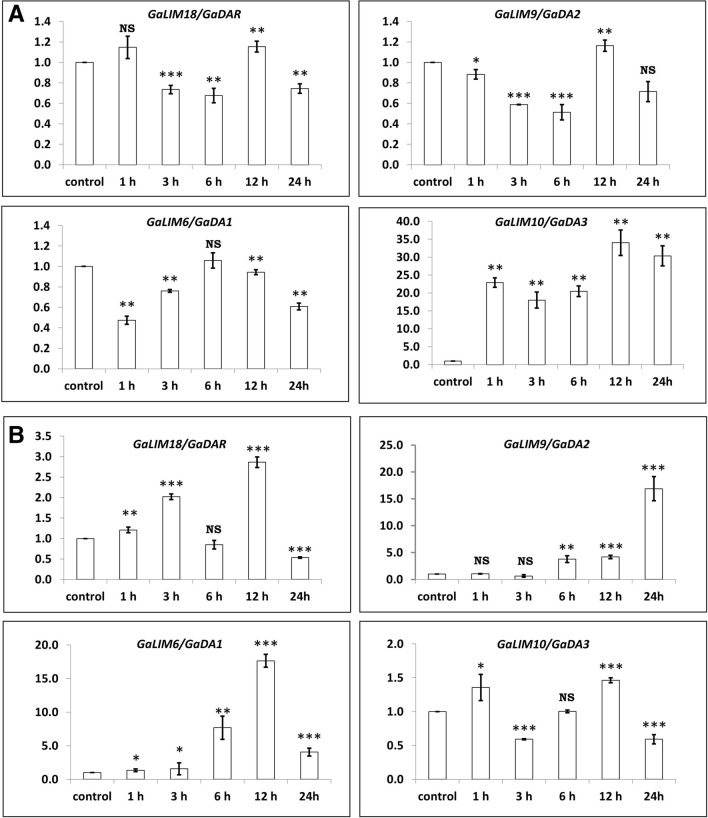
Relative expressions of plant-specific *LIM* family genes after ABA (**a**) and JA (**b**) treatment.

Plant defense hormones such as ABA and JA were sprayed on the 3 weeks old seedlings of cotton. The response of plant-specific *LIM* gene members against these hormones were quantified at transcript level using quantitative real-time PCR (qRT-PCR). Analysis of qRT-PCR data revealed that upon ABA treatment, only *GaDA3/GaLIM10* gene showed significant and steady up-regulation in its expression, whereas, the others *viz. GaDAR1*, *GaDA1* and *GaDA2* genes exhibited variable expression patterns with respect to time. *GaDAR1* and *GaDA2* showed significant down-regulation at 3h and 6h but significant up-regulation of expression at 12h time, followed by significant and non-significant down-regulation of expression at 24h, respectively with respect to control. *GaDA1* showed significant down-regulation of expression as compared to control (Fig. 5a). In case of JA treated seedlings, *GaDA1* and *GaDA2* showed significant up-regulation in expressions after 6h of the treatment as compared to control. Except at 3h, both the genes followed similar expression pattern at different point of time in response to JA treatment. On the other hand, *GaDA3* and *GaDAR1* showed variable expression at different time intervals upon JA treatment (Fig. 5b).

#### Salt treatment

**Figure 6:**
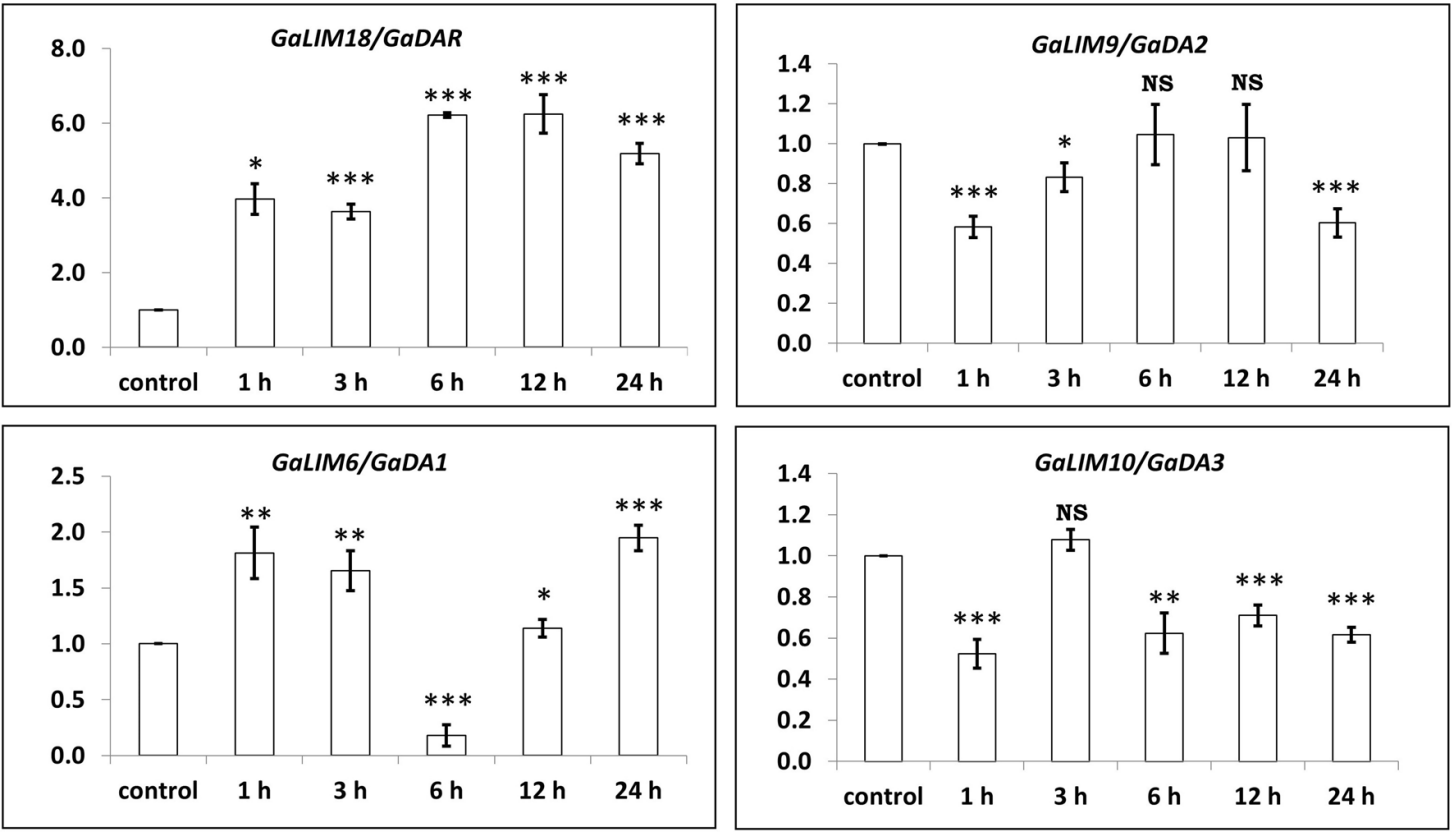
Relative expressions of plant-specific *LIM* family genes in response to salt treatment.

Relative expressions of genes in response to treatment of 200 mM NaCl and mock treated samples as control was quantified. The response of *GaDAR1* to the salt treatment was significant with elevated transcript level in comparison to mock treated samples. *GaDA1* showed significant up-regulation at early induction periods (1hr and 3hrs) but steep decline in the expression was noticed at 6h, before significant up-regulation at 12h and 24h of time respectively. In contrast, significant decline in transcript levels of *GaDA2* and *GaDA3* in response to salt treatment was noticed (Fig. 6).

#### Biotic stress

**Figure 7:**
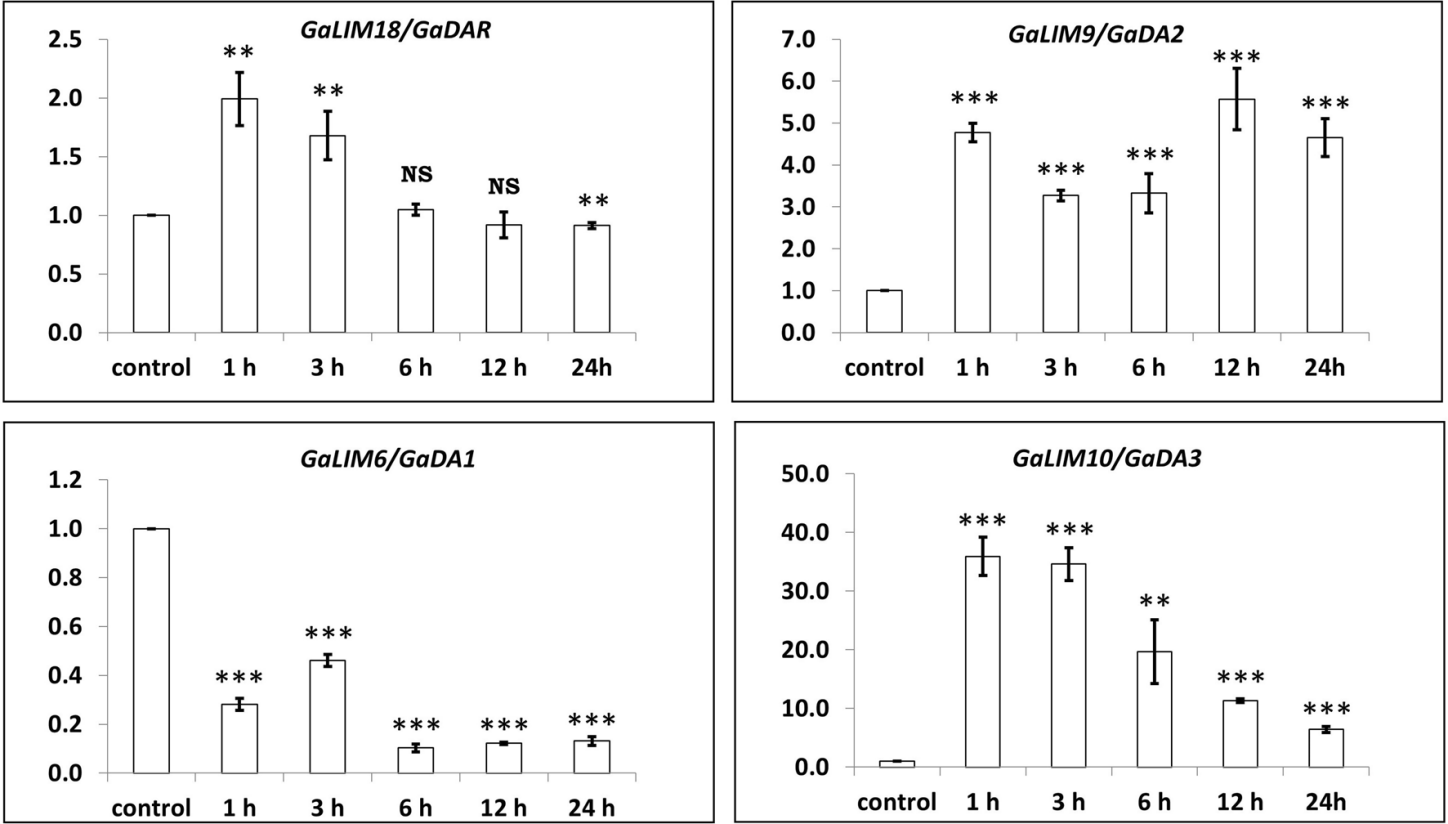
Relative expressions of plant-specific *LIM* family genes in response to challenge of *Fusarium oxysporum*. NB Relative gene expression levels are represented with respect to their expressions in mock treated control plants. Gene expression in control was used as calibrator (set to unit one). Data were normalized using the average expression levels of *Ghactin4* as reference gene. Error bars represent standard deviation. Asterisk denotes significant differences (*P > 0.05, **P > 0.01 and ***P > 0.001) compared to their respective controls; *NS* non-significant.

Expression of *LIM* genes in response to challenge inoculation of *F. oxysporum* resulted in identifying *GaDA2* as putative candidate gene as it provided significant up-regulation across time intervals. *GaDAR1* and *GaDA3* showed induced responses during early hours of treatment, but significant transcript level reductions were observed for both at 24h of treatment (Fig. 7). Upon treatment, expression of *GaDA1* was significantly down-regulated.

## Discussion

Biologically, cotton fibre is a single elongated epidermal cell whose development involves initiation, elongation, secondary cell wall synthesis and maturation phases. Its quality features such as fibre length, strength and fineness are resultants of interactions between cellulosic, non-cellulosic components along with network of cytoskeleton protein components. Fibre strength is strongly influenced by the thickness of the secondary cell wall and the ultra-structure of the cellulose^50,51^. A number of actin-binding proteins are known to play pivotal role in fibre development from the elongation stage to the secondary wall synthesis stage^29,52^. Among various such actin-binding proteins, LIM domain containing protein families have recently been identified as potential regulators of actin dynamics and cytoskeleton reorganization in several plants species. Several LIM proteins *viz.* GhWLIM1a, GhWLIM5, GhPLIM1 and GhXLIM6 were characterized for their roles in fibre and pollen development of upland cotton^16,18,31,32^. Except those few reports, a comprehensive understanding of structure and function of LIM genes in cotton is still in its infancy. Hence, we attempted to unravel LIM domain proteins of diploid *G. arboreum* using genomic information available in public domain. Unless mentioned otherwise, this is the first report on comprehensive genome-wide characterization of *LIM* genes from *G. arboreum.* In the present study, 20, 18 and 35 *LIM* genes were identified in *G. arboreum*, *G. raimondi* and *G. hirsutum*, respectively. This disparity between the number of *LIM* genes among those three species might have resulted from differential selection pressure occurred during the course of evolution and/or errors associated with assembling of whole genome sequencing data^39^.

The identified twenty candidate LIM proteins from *G. arboreum*, contained either one or two LIM domain/s, distributed across the genome. The total number of LIM proteins identified from *G. arboreum* (twenty) was more than those reported in *A. thaliana* (fourteen), *P. trichocarpa* (twelve) and *C. arietinum* (fifteen). Differences in the number of *LIM* genes between different plant genera might be due to the differential expansions of gene family occurred during the course of their evolution. For instance, at around 93 million years ago (mya), *G. arboreum* shared a common ancestry with *Arabidopsis*. During the course of evolution, both had undergone subsequent cycles of whole genome duplications resulting in variable expansion of several genes families^34,53,54^. Evidently, our gene duplication analysis revealed that around 85% of the entire *LIM* gene family in *G. arboreum* showed predominantly segmental paralogous gene duplication which might be instrumental in the expansion of *GaLIM* gene family. This is supported by our results indicating an approximate evolutionary timeline of 1.81 to 28.57 mya for gene duplication event of *GaLIM*s (Supplementary Table S7), coinciding with recent whole genome duplication of *G. arboreum* which occurred around 13 to 20 mya^34^. In phylogeny, these proteins were distinctly categorized into two sub-families (2LIM and plant-specific DA1/DAR) based on their number of LIM domains and their sub-division was also supported by high bootstrap values (Fig. 3). Similar categorization of *LIM* gene family members was also deduced in different monocot and dicot plant families such as Brassicaceae, Solanaceae, Poaceae, Malvaceae and Fabaceae suggesting their conserved evolutionary relatedness^7,11,12,14,15,17,18,24^.

The first subfamily, called 2LIM (containing two LIM domains) sharing features with animal CRPs, harbours sixteen *GaLIM*s, while the other four *GaLIM*s were clustered with plant-specific DA/DAR subfamily. Interestingly, in our study, out of 16 CRP-like *GaLIM*s, only 11 LIMs had features of 2LIM domain, while remaining five LIM proteins were predicted to possess single LIM domain (Supplementary Fig. S1). This finding is in contrast to all the earlier reports which suggest that plant LIM proteins sharing structural analogy with animal CRPs typically contain two LIM domains separated by a spacer of around 50 amino acids^55^. Therefore, our study provided a further sub-division of animal CRP-like LIMs in plants into two subgroups, representing both single and double LIM domain containing proteins based upon their domain number/s. In plants, only single member of *Brassica* LIM family [*Br*WLIM2b (*Br*LIM6)] is reported to possess single LIM domain similar to CRP-like LIM^12^. The role of LIM domain in plants has not been clearly understood. However, among the fewer characterized *LIM* genes, they are known to be associated with the transcription regulation of phenyl-propanoid pathway genes involved in secondary cell wall biosynthesis and pollen tube development, apart from their implicit role in actin dynamics^11,16,18,56^. Hence, it will be interesting to explore and functionally validate CRP-like 1LIM domain containing proteins in other plants including cotton. The available whole genome information of various plants may be explored for deriving deeper insights on plant LIMs.

There are subgroups of αLIM1, βLIM1, γLIM2 and δLIM2 which exhibit tissue and/or stage specific functions^7^. In the present study, expression pattern and phylogenetic analysis confirmed the presence of four distinct LIM subgroups *viz*. FLIM1/XLIM1, WLIM1, WLIM2 and PILM2 distributed within αLIM1, βLIM1, γLIM2 and δLIM2 (Figs. 3 and 4). Absence of subgroup PLIM1 was observed which is in concurrence with the other plant species such as *A. thaliana*, *O. sativa*, *P. trichocarpa*, *C. arietinum*, *B. rapa*^7,12,14^. δLIM2 group formed a large group with 8 out of 16 GaLIM members. In Arabidopsis, members of PLIM2 group (*AtPLIM2a*-*2c*) were reported to be highly expressive in reproductive tissues particularly in pollen and are functionally linked to actin dynamics^57^. Similarly, members of *PLIM2* (*PtPLIM2a*) from Poplar had shown remarkably elevated expressions in matured anthers^7^. In our study, four *GaLIMs* (*GaPLIM2a*-*2d*) formed clusters with PLIM2 group members belonging to *A. thaliana*, *P. trichocarpa* and *C. arietinum* (Fig. 3). Moreover, *GaPLIM2a* and *GaPLIM2c* had shown preferential expressions in anthers which suggested a functional analogy of *GaPLIMs* akin to that of *A. thaliana*, *P. trichocarpa* and *C. arietinum* (Fig. 4). These observations are also supported by the fact that PLIM2 exhibited profound transcript abundance in pollen development phase in majority of eudicots^7^. The other two *GaPLIMs* (*GaPLIM2b* and *2d*) sharing close similarity with *GaPLIM2a* and *2c*, were also expressed in anther tissue. In addition, they also showed expressions in cotton fibre development stages indicating their dual functions in relation to pollen and cotton fibre development. This is in congruence with the earlier studies on expression patterns in Poplar (*PtPLIM2a* and *2b*)^24^. Gene duplication (tandem and/or segmental) is considered to be a key driving force for expansion of gene families. Moreover, it is possible that the duplicated gene members may retain their original function or undergo pseudogenization, subfunctionalization and neofunctionalization process of evolution^40,64^.

Another sub-clade of δLIM (GaδLIM2/*Ga*PLIM2-like) distantly related to PLIM2 subgroup was also observed in the phylogeny of *GaLIM*s. During evolutionary expansion of Asterids δLIM, the members had diverged following PLIM2 gene duplication and formed a separate monophyletic clade namely PLIM2-like (earlier δLIM2), distinct from previously identified PLIM2 members from Tobacco (*NtPLIM2*) and Sunflower (*HaPLIM2*)^7,24^. However, beside differences in sequence identity, the members of PLIM2-like were also found to be strongly expressed in pollen. The phylogenic and expression analysis revealed that four GaLIMs (GaδLIM2a-2d) were clustered with *P. trichocarpa* δLIMs (PtδLIM2a and 2b) (Figs. 3 and 4). The expression specificities of *GaδLIM2* genes suggested a functional conservation during their evolutionary divergence from parental GaPLIM2. The expression patterns of members of GaδLIM2 have also indicated their potential roles during pollen development and cotton fibre development. Interestingly, δLIM2 members are also known to perform dual functions as actin modulators during pollen development and cottony hair formation along with other functions in leaf and vascular tissues^24^.

GaWLIM1 members showed ubiquitous nature of expression pattern with a tendency of transcript abundance particularly during cotton fibre developmental stage (Fig. 4). Several reports have elucidated the roles of *WLIM1* including *F/G/XLIM*s genes during lignin biosynthesis and secondary cell wall biosynthesis via modulating the actin dynamics and expression patterns of PAL box genes involved in phenyl propanoid pathway^58^. In *P. trichocarpa*, *PtWLIM1a* and *PtWLIM1b* are found to be associated with lignin biosynthesis during secondary xylem formation^24^. In the present study, three *GaLIM*s (*GaWLIM1, GaFLIM1a* and *1b*) showed close similarity with poplar WLIM1 members (Fig. 3), thus suggesting a possible similarity of *GaWLIM1* in actin modulating feature as well. This is further supported by the studies in upland cotton where the activity of GhLIMs (*GhXLIM6, GhWLIM1a* and *GhWLIM5*) was reported to play regulatory roles in cotton fibre development stage via interacting with F-actin dynamics and acting as negative regulators of fibre development^16,58^. Another distinct GaLIM (*GaβLIM1a*) had also expressed in all the tissues of *G. arboreum* including early fibre development stage corresponding to 10 DPA (Fig. 4). Despite exhibiting similar pattern of gene expression like GaWLIM1 subgroup, it formed a separate cluster distinct from *GaWLIM1* owing to its sequence divergence and grouped with βLIM members of poplar and chickpea in phylogeny, hence designated as *GaβLIM1a*. βLIM expression in different tissues of poplar^7^ and chickpea^14^ supported this classification and nomenclature.

Apart from sixteen CRP-like LIM proteins, four other plant specific GaLIM (DA1/DAR) were found in *G. arboreum* with presence or absence of UIM and highly conserved C-terminal domain, when compared with that of Arabidopsis and chickpea genomes. Further, phylogenetic analysis classified GaDA1 from *G. arboreum* into three categories *i.e.,* GaDA1, GaDA2 and GaDA3 (Fig. 3). Plant-specific LIMs of DA1 and DAR are known to be involved in biotic and abiotic stress responses and organ size regulation^13,14,22,59^. In our study, plant-specific *GaDA1*/*GaDAR* revealed ubiquitous expression in all tissues. Similarly, in chickpea, majority of *CaDA1* and *CaDAR* members recorded their expressions in most of the tissues and developmental phases. Functional significance of LIM sub-family was investigated in Arabidopsis, where those genes were found to regulate organ size, apoptosis and freezing tolerance^60^, which signifies their roles in biotic and abiotic stresses. Studies in chickpea under biotic and abiotic stresses also drew similar correlation between *DA1/DAR* and stress response^22^. Phylogenetic closeness of *GaDA1*/*DAR* with that of *A. thaliana*, *B. rapa*, *G*. *max* and *C. arietinum* counterparts seems to vindicate our results (Supplementary Fig. S8). Hence, to investigate the role of *GaDA1*/*DAR* under stress, certain abiotic and biotic stresses were imposed. For this, defense hormones application (JA and ABA), salt treatment (NaCl) and wilt causing pathogen (*F. oxysporum*) inoculation were used to understand the response of *GaDA1/DAR* genes. Expressions of all the members of *GaDA1/DAR* family were altered as compared to control under the influence of stress treatments. Among the notable changes, expression of *GaDA1* of ABA treated and pathogen inoculated treatments followed almost similar pattern of down-regulation with respect to time and control. On contrary, there was a steady up-regulation of *GaDA2* in the treatments of JA and *F. oxysporum, respectively.* A strong correlation of ABA and JA with pathogenesis of *F. oxysporum* demonstrates the interference of this pathogen with the signaling pathways of ABA and JA^61^. Incidentally, GaDA2 LIM harbors an additional SCOP domain (SCOP d1i9za), at amino acid sequence position 19 to 158 which belongs to exonuclease-endonuclease-phosphatase (EEP) domain superfamily. EEP superfamily of proteins contains catalytic domain with characteristic feature of phosphodiester bonds cleavage activity, with their substrates being nucleic acids, phospholipids and some proteins as well^62^. All these features are therefore, relatable to a typical component of cell repair mechanism which often gets activated under stress. This probably justifies the designation of *GaDA2* as a candidate gene for *F. oxysporum* related to biotic stress in *G*. *arboreum* cotton. Apart from this, DA1 also contains Ubiquitin Interacting Motif (UIM) that may participate in ubiquitination process leading to several crucial cellular phenomena like nucleotide repair and stress response^63^. The role of DAR LIM members in biotic and abiotic stress response have been vividly demonstrated in *B. rapa*^12^ and Arabidopsis^60^, respectively. In view of the variable responses of *GaDAR* under different abiotic and biotic stresses in cotton, further functional validation studies are required to ascertain their exact role in stress response.

## Conclusion

This comprehensive study exploring whole genome information of *G. arboreum* has revealed twenty LIM domain containing proteins and categorized them on the basis of gene expression patterns and phylogenetic relationships into distinct groups. The study has identified a novel group of animal CRP-like GaLIM proteins containing only single LIM domain with a unique Zn-finger motif architecture. This study is incidentally the first of its kind in cotton and second in plants after *Brassica*. Present study also expands a scope for detailed understanding of roles of each GaLIM proteins corresponding to their LIM domain organizations in plants. Moreover, quantitative gene expression studies have unveiled plant-specific *GaDA2* as a candidate gene under stress response in *G. arboreum*. These findings can be further validated and applied to develop a potential genetic marker for cotton breeding program. This study sets benchmark parameters to search for new LIM-domain proteins from unexplored genomes of *Gossypium* species*.*

## Supplementary Information

Supplementary Information 1.

Supplementary Information 2.
