# Supplementary material for: Genome-wide identification and expression analysis of the plant specific LIM genes in Gossypium arboreum under phytohormone, salt and pathogen stress
Source: Sci Rep. 2021 Apr 28;11:9177. doi: 10.1038/s41598-021-87934-0 (PMC8080811; doi:10.1038/s41598-021-87934-0)

**Supplementary Material**

**Title: Genome-wide identification and expression analyses of the plant specific LIM genes in *Gossypium arboreum* under phytohormone, salt and pathogen stress**

**Author names and affiliation:Raghavendra KP**^1*¥^, Joy Das^1¥^, Rakesh Kumar^1¥^, Shailesh P Gawande^2^, Santosh HB^1^, Annie Sheeba J^3^, S Kranthi^2^, Kranthi KR^4^,Waghmare,V.N^1^

^1^*Division of Crop Improvement, ICAR – Central Institute for Cotton Research (CICR), Nagpur (Maharashtra), INDIA;*

^2^*Division of Crop Protection, ICAR – Central Institute for Cotton Research (CICR), Nagpur (Maharashtra), INDIA*

^3^*Division of Crop Production, ICAR – Central Institute for Cotton Research (CICR), Nagpur (Maharashtra), INDIA;*

^4^*Technical Information Section, International Cotton Advisory Committee (ICAC), Washington DC, USA.*

***E-mail address of the corresponding author:** [kpraghavendra@gmail.com](mailto:kpraghavendra@gmail.com)

**Supplementary Table S1:** qPCR primers for the *GaLIM* gene family

| **S. No.** | **Primer Name** | **Sequence(5'-3')** | **Amplicon length (bp)** |
| --- | --- | --- | --- |
| 1 | LIM A_13549F | AACTGACAAGATCGCCTAGC | 111 |
|  | LIM A_13549R | GACCTTCCACTGTTACCTTCTC |  |
| 2 | LIM A_00967F | TAACTCCCGAGCTGACAAGA | 219 |
|  | LIM A_00967R | TCCTTCCACCGTTACCTTCT |  |
| 3 | LIM A_21476F | AAACAGCGAAGACCGAGAAG | 98 |
|  | LIM A_21476R | CTCGCAAGCAGCACATTTATC |  |
| 4 | LIM A_14459F | CAGACAAAGACTCCGAACAGAC | 106 |
|  | LIM A_14459R | CCTCCACTGTCACCTTCTCTAA |  |
| 5 | LIM A_31990F | TGAATAGGACTCCCAGCAAAC | 122 |
|  | LIM A_31990R | CTTGTGGAAACATTCACCTTCC |  |
| 6 | LIM A_09995F | GTATTGCAAGCCACACTTTGAG | 103 |
|  | LIM A_09995R | AGCCTTTCCCGTTCCATTC |  |
| 7 | LIM A_23003F | GATGAGCACTTACTCCTCCATAG | 113 |
|  | LIM A_23003R | TGGGTCTTAAACCTGTTAGGAAA |  |
| 8 | LIM A_26589F | CTCCTTTGAGGGTGTCTTGTAT | 118 |
|  | LIM A_26589R | AGCTGATCGGTCTACCCTAA |  |
| 9 | LIM A_06392F | GTTGTCAGACCTGAAAGACAAATC | 131 |
|  | LIM A_06392R | CCATTCACAGTAACCCTCTCAA |  |
| 10 | LIM A_21729F | CTGGCAGTCTAGACAAGAGTTT | 92 |
|  | LIM A_21729R | ATTCACCACTTTCTGTGCATTC |  |
| 11 | LIM A_22981F | CTTCAAGGAGACGGGTAACTTC | 93 |
|  | LIM A_22981R | TTGCTTGGTGATCTCGTCAG |  |
| 12 | LIM A_14838F | GTTGATGGTGAGAAACCAATAGC | 119 |
|  | LIM A_14838R | GTACCATTCACTGTAACCCTCTC |  |
| 13 | LIM A_02312F | CAAAGGGCACCAAGCAAAG | 109 |
|  | LIM A_02312R | TTTCACCTTCCACAGTCACC |  |
| 14 | LIM A_32215F | TTTCTGCAATCACGAGACCTT | 84 |
|  | LIM A_32215R | AGCTGGGAGAAGTGATGTTTAC |  |
| 15 | LIM A_29508F | GATCAGGTGAAGACTCCAAACA | 126 |
|  | LIM A_29508R | ATGGGCGTAGTTGCCTTT |  |
| 16 | LIM A_11634F | GTTCAAAGAGAAAGGCAGCTATG | 98 |
|  | LIM A_11634R | CCTCTGGTGTTGGATCATCTT |  |
| 17 | LIM A_13720F | GTCATCGTCTTCGAGCAGTAG | 101 |
|  | LIM A_13720R | GGGAAGCATCTGATTCGATTTG |  |
| 18 | LIM A_33035F | TACGCAGCTCGATTCTTCTTC | 107 |
|  | LIM A_33035R | CACCCAGCTTCCTCTCAAAT |  |
| 19 | LIM A_24851F | CCGGAAGTTGAGGAAGGAATATG | 107 |
|  | LIM A_24851R | GATGACGAGGATGACGATGAAG |  |
| 20 | LIM A_25109F | AGGAGGCCGACAACTTATTG | 101 |
|  | LIM A_25109R | GCAGTCTTGGAAGACCATACA |  |
| 21 | Actin F RTPCR | GAAGGATATGCCCTTCCACATG | 370 |
|  | Actin R RTPCR | GATATCCACATCGCACTTCATG |  |
| 22 | qGhActin4 F | TTGCAGACCGTATGAGCAAG | 105 |
|  | qGhActin4 R | ATCCTCCGATCCAGACACTG |  |

**Supplementary Table S2:**Pairwise amino acid sequence similarity index (%) among twenty GaLIMs

| **GaLIMs** | **GaLIM1/GaδLIM2a** | **GaLIM2/GaPLIM2b** | **GaLIM3/GaPLIM2a** | **GaLIM4/GaPLIM2c** | **GaLIM5/GaβLIM1a** | **GaLIM6/GaDA1** | **GaLIM7/GaδLIM2b** | **GaLIM8/GaδLIM2c** | **GaLIM9/GaDA2** | **GaLIM10/GaDA3** | **GaLIM11/GaWLIM2b** | **GaLIM12/GaWLIM1a** | **GaLIM13/GaFLIM1a** | **GaLIM14/GaWLIM2c** | **GaLIM15/GaWLIM2d** | **GaLIM16/GaδLIM2d** | **GaLIM17/GaFLIM1b** | **GaLIM18/DAR** | **GaLIM19/GaPLIM2d** |
| --- | --- | --- | --- | --- | --- | --- | --- | --- | --- | --- | --- | --- | --- | --- | --- | --- | --- | --- | --- |
| **GaLIM2/GaPLIM2b** | 52.47 |  |  |  |  |  |  |  |  |  |  |  |  |  |  |  |  |  |  |
| **GaLIM3/GaPLIM2a** | 61.17 | 79.31 |  |  |  |  |  |  |  |  |  |  |  |  |  |  |  |  |  |
| **GaLIM4/GaPLIM2c** | 56.31 | 81.21 | 79.26 |  |  |  |  |  |  |  |  |  |  |  |  |  |  |  |  |
| **GaLIM5/GaβLIM1a** | 52.46 | 52.86 | 50.57 | 48.63 |  |  |  |  |  |  |  |  |  |  |  |  |  |  |  |
| **GaLIM6/GaDA1** | 16.08 | 11.88 | 16.37 | 14.14 | 17.14 |  |  |  |  |  |  |  |  |  |  |  |  |  |  |
| **GaLIM7/GaδLIM2b** | 65.90 | 58.79 | 59.57 | 58.55 | 51.37 | 13.88 |  |  |  |  |  |  |  |  |  |  |  |  |  |
| **GaLIM8/GaδLIM2c** | 63.56 | 41.33 | 54.95 | 54.24 | 41.75 | 13.00 | 58.47 |  |  |  |  |  |  |  |  |  |  |  |  |
| **GaLIM9/GaDA2** | 18.50 | 12.50 | 16.28 | 13.54 | 17.14 | 85.38 | 14.76 | 13.86 |  |  |  |  |  |  |  |  |  |  |  |
| **GaLIM10/GaDA3** | 18.81 | 12.96 | 16.09 | 13.92 | 16.57 | 69.51 | 15.09 | 12.62 | 68.35 |  |  |  |  |  |  |  |  |  |  |
| **GaLIM11/GaWLIM2b** | 44.71 | 58.59 | 47.53 | 47.65 | 45.61 | 13.45 | 45.88 | 26.67 | 14.04 | 14.04 |  |  |  |  |  |  |  |  |  |
| **GaLIM12/GaWLIM1a** | 50.81 | 45.77 | 51.41 | 48.11 | 69.15 | 17.14 | 49.73 | 42.86 | 16.57 | 15.43 | 42.20 |  |  |  |  |  |  |  |  |
| **GaLIM13/GaFLIM1a** | 48.94 | 45.14 | 48.59 | 45.99 | 70.37 | 15.93 | 48.42 | 41.35 | 15.93 | 16.48 | 43.86 | 77.66 |  |  |  |  |  |  |  |
| **GaLIM14/GaWLIM2c** | 60.71 | 58.04 | 62.01 | 60.22 | 55.68 | 13.79 | 60.75 | 55.66 | 14.37 | 14.37 | 66.28 | 54.55 | 54.30 |  |  |  |  |  |  |
| **GaLIM15/GaWLIM2d** | 52.46 | 57.65 | 53.51 | 54.92 | 42.74 | 13.39 | 52.46 | 46.24 | 14.29 | 14.29 | 54.70 | 38.10 | 41.94 | 72.58 |  |  |  |  |  |
| **GaLIM16/GaδLIM2d** | 55.25 | 39.26 | 50.00 | 48.60 | 41.40 | 12.20 | 52.20 | 63.56 | 13.33 | 13.17 | 31.94 | 41.51 | 39.87 | 52.50 | 47.12 |  |  |  |  |
| **GaLIM17/GaFLIM1b** | 47.50 | 43.87 | 47.28 | 44.44 | 69.84 | 14.95 | 48.02 | 42.48 | 15.46 | 16.49 | 42.69 | 76.06 | 79.59 | 54.55 | 43.55 | 38.24 |  |  |  |
| **GaLIM18/DAR** | 18.50 | 13.75 | 17.44 | 14.06 | 15.43 | 55.84 | 15.24 | 12.87 | 42.67 | 59.35 | 14.62 | 14.86 | 15.38 | 15.52 | 15.18 | **10.91** | 14.95 |  |  |
| **GaLIM19/GaPLIM2d** | 54.90 | 76.22 | 79.79 | 82.61 | 47.51 | 13.68 | 56.73 | 53.39 | 12.57 | 12.44 | 47.02 | 46.99 | 44.86 | 59.24 | 53.33 | 49.72 | 41.33 | 14.66 |  |
| **GaLIM20/WLIM2a** | 62.90 | 59.44 | 64.61 | 61.83 | 55.91 | 14.94 | 61.83 | 55.66 | 15.52 | 15.52 | 69.36 | 53.72 | 44.55 | **92.02** | 76.00 | 53.12 | 55.61 | 16.09 | 60.87 |

**Supplementary Table S3:**Pairwise amino acid sequence similarity index (%) among sixteen GaLIMs similar to animal Cysteine Rich Proteins (CRPs)

| **GaLIMs** | **GaLIM1/GaδLIM2a** | **GaLIM2/GaPLIM2b** | **GaLIM3/GaPLIM2a** | **GaLIM4/GaPLIM2c** | **GaLIM5/GaβLIM1a** | **GaLIM7/GaδLIM2b** | **GaLIM8/GaδLIM2c** | **GaLIM11/GaWLIM2b** | **GaLIM12/GaWLIM1a** | **GaLIM13/GaFLIM1a** | **GaLIM14/GaWLIM2c** | **GaLIM15/GaWLIM2d** | **GaLIM16/GaδLIM2d** | **GaLIM17/GaFLIM1b** | **GaLIM19/GaPLIM2d** |
| --- | --- | --- | --- | --- | --- | --- | --- | --- | --- | --- | --- | --- | --- | --- | --- |
| **GaLIM2/GaPLIM2b** | 52.47 |  |  |  |  |  |  |  |  |  |  |  |  |  |  |
| **GaLIM3/GaPLIM2a** | 61.17 | 79.31 |  |  |  |  |  |  |  |  |  |  |  |  |  |
| **GaLIM4/GaPLIM2c** | 56.31 | 81.21 | 79.26 |  |  |  |  |  |  |  |  |  |  |  |  |
| **GaLIM5/GaβLIM1a** | 52.46 | 52.86 | 50.57 | 48.63 |  |  |  |  |  |  |  |  |  |  |  |
| **GaLIM7/GaδLIM2b** | 65.90 | 58.79 | 59.57 | 58.55 | 51.37 |  |  |  |  |  |  |  |  |  |  |
| **GaLIM8/GaδLIM2c** | 63.56 | 41.33 | 54.95 | 54.24 | 41.75 | 58.47 |  |  |  |  |  |  |  |  |  |
| **GaLIM11/GaWLIM2b** | 44.71 | 58.59 | 47.53 | 47.65 | 45.61 | 45.88 | 26.67 |  |  |  |  |  |  |  |  |
| **GaLIM12/GaWLIM1a** | 50.81 | 45.77 | 51.41 | 48.11 | 69.15 | 49.73 | 42.86 | 42.2 |  |  |  |  |  |  |  |
| **GaLIM13/GaFLIM1a** | 48.94 | 45.14 | 48.59 | 45.99 | 70.37 | 48.42 | 41.35 | 43.86 | 77.66 |  |  |  |  |  |  |
| **GaLIM14/GaWLIM2c** | 60.71 | 58.04 | 62.01 | 60.22 | 55.68 | 60.75 | 55.66 | 66.28 | 54.55 | 54.30 |  |  |  |  |  |
| **GaLIM15/GaWLIM2d** | 52.46 | 57.65 | 53.51 | 54.92 | 42.74 | 52.46 | 46.24 | 54.70 | 38.10 | 41.94 | 72.58 |  |  |  |  |
| **GaLIM16/GaδLIM2d** | 55.25 | 39.26 | 50.00 | 48.60 | 41.40 | 52.20 | 63.56 | **31.94** | 41.51 | 39.87 | 52.50 | 47.12 |  |  |  |
| **GaLIM17/GaFLIM1b** | 47.50 | 43.87 | 47.28 | 44.44 | 69.84 | 48.02 | 42.48 | 42.69 | 76.06 | 79.59 | 54.55 | 43.55 | 38.24 |  |  |
| **GaLIM19/GaPLIM2d** | 54.90 | 76.22 | 79.79 | 82.61 | 47.51 | 56.73 | 53.39 | 47.02 | 46.99 | 44.86 | 59.24 | 53.33 | 49.72 | 41.33 |  |
| **GaLIM20/WLIM2a** | 62.90 | 59.44 | 64.61 | 61.83 | 55.91 | 61.83 | 55.66 | 69.36 | 53.72 | 44.55 | **92.02** | 76.00 | 53.12 | 55.61 | 60.87 |

**Supplementary Table S4:** Pairwise amino acid sequence similarity index (%) among four plant specific GaLIMs (GaDA1/GaDAR)

| **Plant specific GaLIMs** | **GaLIM6/GaDA1** | **GaLIM9/GaDA2** | **GaLIM10/GaDA3** |
| --- | --- | --- | --- |
| **GaLIM9/GaDA2** | **85.38** |  |  |
| **GaLIM10/GaDA3** | 69.51 | 68.35 |  |
| **GaLIM18/DAR** | 55.84 | **42.67** | 59.35 |

**Supplementary Table S5.Conserved LIM domain sequences identified in *G. hirsutum***

| **Gene ID** | **Length of AAs** | **LIM domain Start - end (aa)** |
| --- | --- | --- |
| Ghi_A13G00891  A13:2006693:2008442 | 189 | 9-61- KCKACQKTVYPVELLSADGIPYHKSCFKCSHCKGTLKLGNYSSMEGVVYCKPH  107-159-KCATCGKTAYPLEKVTVEGQSYHKSCFKCSHGGCPITPSNYAALEGILYCKHH |
| Ghi_D13G00866  D13:1592895:1594642 | 189 | 9-61-KCKACEKTVYPVELLSADGIPYHKSCFKCSHCKGTLKLGNYSSMEGVVYCKPH  107-159-KCATCGKTAYPLEKVTVEGQSYHKSCFKCSHGGCPITPSNYAALEGILYCKHH |
| Ghi_D10G12816  D10:64983563:64985681:- | 188 | 9-61-KCKACEKTVYPVELVSVDGVPFHKSCFKCSHCKGTLKWGNYSSMEGVLYCKPH  106-158-KCATCGKTAYPLEKVTVEGQSYHKSCFKCSHGGCPISPSNYAALEGILYCKHH |
| Ghi_A10G14046  A10:119697617:119699737:- | 189 | 9-61-KCKACEKTVYPVELVSVDGVPFHKSCFKCSHCKGTLKWGNYSSMEGVLYCKPH  106-158-KCATCGKTAYPLEKVTVEGQSYHKSCFKCSHGGCPISPSNYAALEGILYCKHH |
| Ghi_D06G10131  D06:61815973:61817925:+ | 189 | 9-61-KCKACEKTVYPVELLSADGVPYHKSCFKCSHCKGTLKLANYSSMEGVLYCKPH  107-159-KCATCGKTAYPLEKVTVEGQSYHKSCFKCSHGGCSLSPSNYAALEGILYCKHH |
| Ghi_D12G07496  D12:45489208:45490386:+ | 208 | 9-61-KCKACDKTVHVVDMMTLEGVPYHKTCFKCSHCKGNLQMTTYSWMDGILYCKPH  102-154-KCAACEKTVYPLEKVTMEGECFHKTCFRCAHGGCPLTHSSYAALDGVLYCKHH |
| Ghi_A06G01216  A06:2691234:2693323:+ | 190 | 9-61-KCMACDKTVYLVDKLTADNRVYHKACFRCHHCKGTLKLGNYNSFEGVLYCRPH  109-161-KCVGCKNTVYPTERVTVNGTPYHKSCFKCTHGGCVISPSNYIAHEGRLYCKHH |
| Ghi_A12G06686  _A12:82473560:82474730:+ | 208 | 9-61-KCKACDKTVHVVDMMTLEGVPYHKTCFKCSHCKGNLQMTTYSWMDGILYCKPH  102-154-KCAACEKTVYPLEKVTMEGECFHKTCFRCAHGGCPLTHSSYAALDSVLYCKHH |
| Ghi_D02G11391  D02:68783717:68785152:- | 185 | 2-51-ACDKTVYLVDKLTADNRVFHKACFRCHHCKGTLKLSNYNSFEGVLYCRPH  97-149-KCVGCNKTVYPIEKVTVDGTSYHRGCFKCSHGGCTISPSNYVAHEGKLYCKHH |
| Ghi_D06G01081  D06:2396393:2398478:+ | 190 | 9-61-KCMACDKTVYLVDKLTADNRVYHKACFRCHHCKGTLKLGNYNSFEGVLYCRPH  109-161-KCFGCKNTVYPTERVTVNGTPYHKSCFKCTHGGCVISPSNYIAHEGRLYCKHH |
| Ghi_D11G05071  D11:9122564:9123535:+ | 209 | 9-61-KCKACDKTVHVVDMMTLEGVPYHKTCFKCSHCKGNLVMNTYSSMDGVLYCKPH  104-156-KCAACEKTVYPLEKITMEGECFHKTCFRCAHGGCPLTHSSYAALNGILYCKHH |
| Ghi_D04G0917  D04:54117239:54119195:- | 208 | 10-62-KCNACNKTVYLVDKLTADNRVFHKACFRCHHCKGTLKLSNYNSFEGVLYC  108-160-KCAACSKTAYPIERVTVNGTIYHKSCFKCTHGGCTISPSNYIAHEGKLYCKHH |
| Ghi_A04G06521  A04:82113838:82116101:- | 208 | 10-62-KCNACNKTVYLVDKLTADNRVFHKACFRCHHCKGTLKLSNYNSFEGVLYCRPH  108-160-KCAACSKTAYPIERVTVNGTIYHKSCFKCTHGGCTISPSNYIAHEGKLYCKHH |
| Ghi_A11G04726  A11:8488983:8489953:+ | 209 | 9-61-KCKACDKTVHVVDMMTLEGVPYHKTCFKCSHCKGNLVMNTYSSMDGVLYCKPH  104-156-KCAACEKTVYPLEKITMEGECFHKTCFRCAHGGCPLTHSSYAALNGILYCKHH |
| Ghi_A08G08411  A08:101231374:101232709:+ | 207 | 9-61-KCKACDKTVHVVDMLTLEGVPYHKTCFKCSHCKGNLVMSTYSSMDGVLYCKPH  103-155-KCAACHKTVYPLEKVTMEGECFHKTCFRCAHGGCALTHSSYAALDGVLYCKHH |

| Ghi_D08G08016  D08:48387509:48388925:+ | 207 | 9-61-KCKACDKTVHVVDMLTLEGVPYHKTCFKCSHCKGNLVMSTYSSMDGVLYCKPH  103-155-KCAACHKTVYPLEKVTMEGECFHKTCFRCAHGGCALTHSSYAALDGVLYCKHH |
| --- | --- | --- |
| Ghi_D08G01886  D08:3824843:3827760:- | 193 | 9-61-KCNACDKTVHVVDMLTLEGIPYHKTCFKCSHCKGNLVMSTYSSMDGVLYCKPH  96-148-KCASCEKTVYPLEKVTIEGECFHKSCFKCAHGGCHLTHSSYAALNGVLYCKHH |
| Ghi_A12G13276  A12:106906956:106908005:- | 225 | 9-61-KCTICDKTVHFIDLLTADGIPYHKTCFKCTHCNGLLVMSNYCSMEGVLYCKPH  104-156-KCGVCNKTAYPLEKVTVEGENYHKSCFRCSPGGCLLTPSTYAAMDGILYCKHH |
| Ghi_D08G10666  D08:58232258:58233063:- | 217 | 9-61-KCKACDKTVHVIDLLTADGISYHKTCFKCSHCHGVLVMGNYCSMDGVLYCKPH  104-156-KCGVCKKTAYPLEKVTVEGENYHKSCFRCSHGGCVLTPSTYAALEGFLYCKHH |
| Ghi_A12G15831  A12:111566825:111568680:- | 189 | 10-62-KCKACEKTVYLVDQLTADNKVYHKACFRCHHCKGTLKLSNYSSFEGVLYCKPH  108-160-KCVACKKTVYPIEKVAVDGTSYHKACFRCTHGGCVISPSNYVAHEHRLYCRHH |
| Ghi_D12G16341  D12:68758613:68760428:- | 189 | 10-62-KCKACEKTVYLVDQLTADNKVYHKACFRCHHCKGTLKLSNYSSFEGVLYCKPH  108-160-KCVACKKTVYPIEKVAVDGTSYHKACFRCTHGGCVISPSNYVAHEHRLYCRHH |
| Ghi_A03G11906  A03:113207813:113209313:- | 201 | 10-62-KCMACDKTVYLVDKLTADNRVFHKACFRCHHCKGTLKLSNYNSFEGVLYCRPH  108-160-KCVGCNKTVYPIEKFFFKKVAVDGTSYHRSCFKCSHGGCTISPSNYIAHEGKLYCKHH |
| Ghi_A08G11351  A08:116503107:116503912:- | 217 | 9-61-KCKACDKTVHVIDLLTADGISYHKTCFKCSHCHGVLVMGNYCSMDSVLYCKPH  104-156-KCGACKKTAYPLEKVTIEGENYHKSCFRCSHGGCVLTPSTYAALEGFLYCKHH |
| Ghi_D11G01066  D11:1817165:1818419:- | 202 | 9-61-KCRSCDKTVHFIEMVSVDGVPYHKTCFRCSHCKGLLVMGSHCQREGNLYCKPH  98-150-KCGVCKKTCYPLEKVTVEGEIYHKNCFRCAHGGCFLTTSSYAALDGFLYCKHH |
| Ghi_A11G01191  A11:2096545:2097779:- | 206 | 9-61-KCRSCDKTVHFIEMVSVDGVPYHKTCFRCSHCKGLLVMGSHCQREGNLYCKPH  102-154-KCGVCKKTCYPLEKVTVEGEIYHKNCFRCAHGGCFLTTSSYAALDGFLYCKHH |
| Ghi_D12G13821  D12:64271137:64272161:- | 217 | 9-61-KCTVYDKTVHFVDLLTADGIPYHKTCFKCTHCNGLLVMSNYCSMEGVLYCKPH  104-156-KCGVCNKTAYPLEKVTVEGENYHKSCFRCSPGGCLLTPSTYAAMDGILYCRHH |
| Ghi_A08G01906  A08:4161129:4164429:- | 129 | 33-84-TCFKCSHCKGNLVVSMEGECFHKSCFKCAHGGCHLTHSSYAALNGVLYCKHH |
| Ghi_A06G10496  A06:121386989:121394093: | 317 | 235-287-KCATCGKTAYPLEKVTVEGQSYHKSCFKCSHGGCSLSPSNYAALEGILYCKHH |
| Ghi_D03G09036  D03:52879307:52879760:+ | 120 | 24-76-KCGVCKKTAYPLEKVTMEGEIYHKNCFRCSHGGCVLTTSSFAALDGILYCKIH |
| Ghi_A03G01221  A03:3477966:3478413:- | 118 | 24-76-KCGVCKKTAYPLEKVTMEGEIYHKNCFRCSHGGFVLTTSSFAALDGILYCKIH |
| Ghi_D10G13461  D10:67362176:67365227:+ | 520 | 155-207-VCAGCHREIGYGNYLGCMGAYFHPNCFRCHSCGYPITEHEFSLSGRDPYHKTC |
| Ghi_A10G13846  A10:118990794:118993860:+ | 520 | 155-207-VCAGCHRDIGYGNYLGCMGAYFHPNCFRCHSCGYPITEHEFSLSGRDPYHKTC |
| Ghi_A01G05801  A01:26041787:26045258:- | 449 | UIM-43-62-HENEEIDRAIALSLLEESQK  UIM-75-94-EEDEQLARAIQESLRFEPPP  LIM-115-190-ICAGCNTEIGHGRFLNCLNAFWHPECFHCHACNFPISDYEIPTNPAGLIEYRAHPFWIQKYCPSHEHDGTPRCCSC |
| Ghi_D05G07266  D05:12940339:12943850:- | 497 | UIM-63-82-HENEEIDRAIALSLLEESQK  UIM-94-113-EDDEQLARAIQESLNFEPPP  LIM-134-186-ICAGCNTEIGCGRFLNCLSAFWHPECFRCHACNLPISDYEFSMSGNYRFHKSC |
| Ghi_A05G12096  A05:23787766:23797701:- | 663 | UIM-228-247-HENEEIDRAIALSLLEESQK  UIM-259-278-EDDEQLARAIQESLNFEPPP  LIM-ICAGCNTEIGCGRFLNCLSAFWHPDCFRCHACNLPISDYEFSMSGNYRFHKSC |

**Supplementary Table S6.Conserved LIM domain sequences identified in *G. raimondii***

| **Gene ID** | **Length of AAs** | **LIM domain Start - end (aa)** |
| --- | --- | --- |
| D5.v1.pred_00018816-RA | 189 | 9-61- KCKACEKTVYPVELLSADGIPYHKSCFKCSHCKGTLKLGNYSSMEGVVYCKPH  107-159- KCATCGKTAYPLEKVTVEGQSYHKSCFKCSHGGCPITPSNYAALEGILYCKHH |
| D5.v1.pred_00012209-RA | 188 | 9-61- KCKACEKTVYPVELVSVDGVPFHKSCFKCSHCKGTLKWGNYSSMEGVLYCKPH  106-158-KCATCGKTAYPLEKVTVEGQSYHKSCFKCSHGGCPISPSNYAALEGILYCKHH |
| D5.v1.pred_00017288-RA | 208 | 9-61- KCKACDKTVHVVDMMTLEGVPYHKTCFKCSHCKGNLQMTTYSWMDGILYCKPH  102-154- KCAACEKTVYPLEKVTMEGECFHKTCFRCAHGGCPLTHSSYAALDGVLYCKHH |
| D5.v1.pred_00025044-RA | 196 | 10-62- KCMACDKTVYLVDKLTADNRVFHKACFRCHHCKGTLKLSNYNSFEGVLYCRPH  108-160- KCVGCNKTVYPIEKVTVDGTSYHRGCFKCSHGGCTISPSNYIAHEGKLYCKHH |
| D5.v1.pred_00036622-RA | 208 | 10-62- KCNACNKTVYLVDKLTADNRVFHKACFRCHHCKGTLKLSNYNSFEGVLYCRPH  108-160- KCAACSKTAYPIERVTVNGTIYHKSCFKCTHGGCTISPSNYIAHEGKLYCKHH |
| D5.v1.pred_00007880-RA | 209 | 9-61-KCKACDKTVHVVDMMTLEGVPYHKTCFKCSHCKGNLVMNTYSSMDGVLYCKPH  104-156- KCAACEKTVYPLEKITMEGECFHKTCFRCAHGGCPLTHSSYAALNGILYCKHH |
| D5.v1.pred_00010592-RA | 207 | 9-61- KCKACDKTVHVVDMLTLEGVPYHKTCFKCSHCKGNLVMSTYSSMDGVLYCKPH  103-155-KCAACHKTVYPLEKVTMEGECFHKTCFRCAHGGCALTHSSYAALDGVLYCKHH |
| D5.v1.pred_00009243-RA | 203 | 9-61- KCKACDKTVHVVDMLTLEGVPYHKTCFKCSHCKGHLVMSTYSSMDGVLYCKPH  104-156- KCASCEKTVYPLEKVTMEGECFHKSCFKCAHGGCHLTHSSYAALNGVLYCKHH |
| D5.v1.pred_00011149-RA | 217 | 9-61- KCKACDKTVHVIDLLTADGISYHKTCFKCSHCHGVLVMGNYCSMDGVLYCKPH  104-156- KCGVCKKTAYPLEKVTVEGENYHKSCFRCSHGGCVLTPSTYAALEGFLYCKHH |
| D5.v1.pred_00015920-RA | 304 | 9-61- KCTVCDKTVHFVDLLTADGIPYHKTCFKCTHCNGLLVMSNYCSMEGVLYCKPH  125-177- KCGVCNKTAYPLEKVTVEGENYHKSCFRCSPGGCLLTPSTYAAMDGILYCKHH |
| D5.v1.pred_00015355-RA | 211 | 10-84- KCKACEKTVYLVDQLTADNKVYHKACFRCHHCKGTLKIVPKTLKPELQMAESDHLYVLNL SNYSSFEGVLYCKPH  130-182- KCVACKKTVYPIEKVAVDGTSYHKACFRCTHGGCVISPSNYVAHEHRLYCRHH |
| D5.v1.pred_00030780-RA | 186 | 9-57- KCMACDKTVYLVDKLTADNRVYHKACLGAITAKLGNYNSFEGVLYCRPH  105-157- KCFGCKNTVYPTERVTVNGTPYHKSCFKCTHGGCVISPSNYIAHEGRLYCKHH |
| D5.v1.pred_00032771-RA | 292 | 9-61- KCKACEKTVYPVELLSADGVPYHKSCFKCSHCKGTLKLANYSSMEGVLYCKPH |
| D5.v1.pred_00008559-RA | 157 | 53-105- KCGVCKKTCYPLEKVTVEGEIYHKNCFRCAHGGCFLTTSSYAALDGFLYCKHH |
| D5.v1.pred_00040452-RA | 120 | 24-76- KCGVCKKTAYPLEKVTMEGEIYHKNCFRCSHGGCVLTTSSFAALDGILYCKIH |
| D5.v1.pred_00017888-RA | 705 | UIM-75-94- EEDEQLAKAIQESLNVESPP  LIM-115167- ICAGCNAEIGHGRYLSCMGSVWHPECFRCHACNQPINDYEFSVSGNRPFHKSC |
| D5.v1.pred_00002142-RA | 561 | UIM-77-96-HENEEIDRAIALSLLEESQK  UIM-108-127-EDDEQLARAIQESLNFEPPP  LIM- 176-228-ICAGCNTEIGCGRFLNCLSAFWHPECFRCHACNLPISDYEFSMSGNYRFHKSC |
| D5.v1.pred_00012313-RA | 519 | LIM-154-206- VCAGCHREIGYGNYLGCMGAYFHPNCFRCHSCGYPITEHEFSLSGRDPYHKTC |

**Supplementary Table S7:**Time of gene duplication for paralogous *GaLIM* gene pairs (DnaSp softwarev5; http://www.ub.edu/dnasp/)

| **S. No.** | **Gene1** | **Gene2** | **Sequence**  **Similarity (%)** | ***Ks*** | **Tt=Ks/2λ** |
| --- | --- | --- | --- | --- | --- |
| 1 | GaLIM14 | GaLIM20 | 92.02 | 0.05 | 1.81 |
| 2 | GaLIM14 | GaLIM15 | 72.58 | 0.30 | 9.91 |
| 3 | GaLIM20 | GaLIM15 | 76.00 | 0.28 | 9.41 |
| 4 | GaLIM4 | GaLIM19 | 82.61 | 0.17 | 5.70 |
| 5 | GaLIM4 | GaLIM3 | 79.26 | 0.24 | 8.03 |
| 6 | GaLIM4 | GaLIM2 | 81.21 | 0.74 | 24.78 |
| 7 | GaLIM7 | GaLIM8 | 58.47 | 0.17 | 5.80 |
| 8 | GaLIM19 | GaLIM3 | 79.79 | 0.27 | 9.15 |
| 9 | GaLIM19 | GaLIM2 | 76.22 | 0.86 | 28.57 |
| 10 | GaLIM1 | GaLIM8 | 63.56 | 0.14 | 4.80 |
| 11 | GaLIM3 | GaLIM2 | 79.31 | 0.57 | 19.12 |
| 12 | GaLIM17 | GaLIM13 | 79.59 | 0.55 | 18.33 |
| 13 | GaLIM17 | GaLIM12 | 76.06 | 0.67 | 22.45 |
| 14 | GaLIM13 | GaLIM12 | 77.66 | 0.34 | 11.30 |
| 15 | GaLIM16 | GaLIM8 | 63.56 | 0.11 | 3.64 |
| 17 | GaLIM11 | GaLIM20 | 69.36 | 0.35 | 11.56 |
| 18 | GaLIM6 | GaLIM9 | 85.38 | 0.13 | 4.43 |


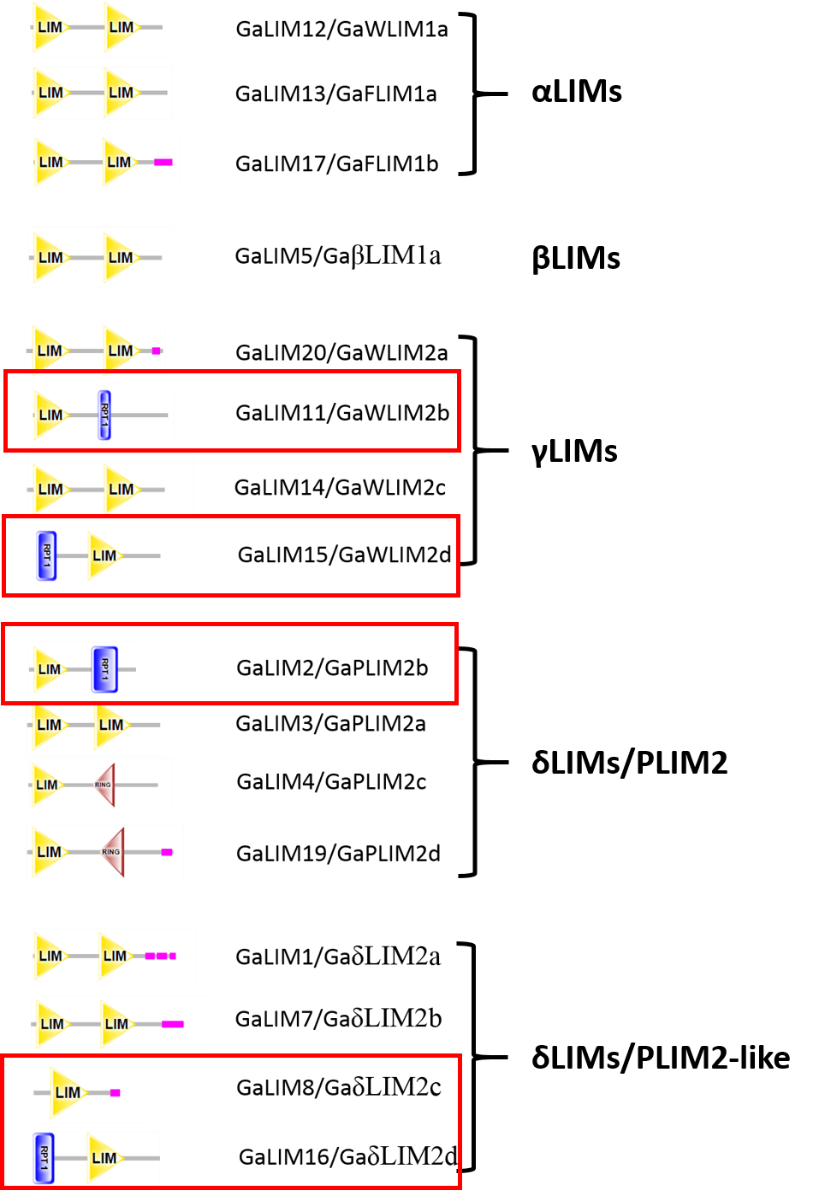


**Supplementary Fig. S1**SMART analysis LIM proteins of *G. arboreum*


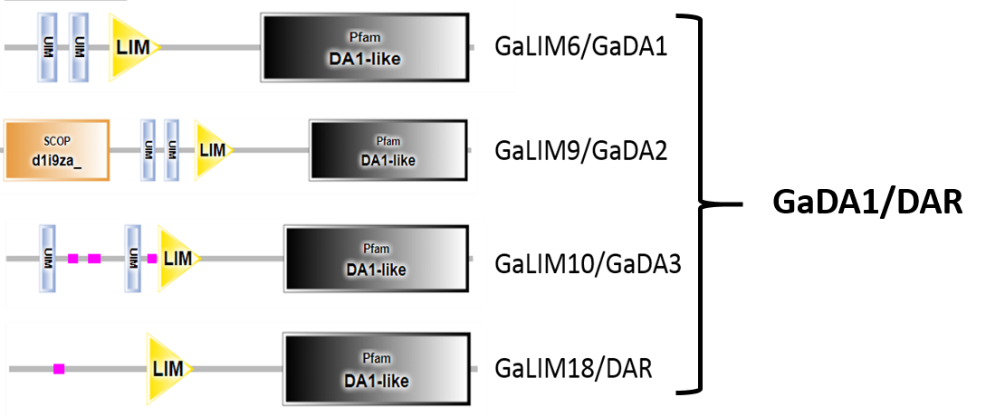


**Supplementary Fig. S2:**SMART analysis plant-specific LIM proteins of *G. arboreum*


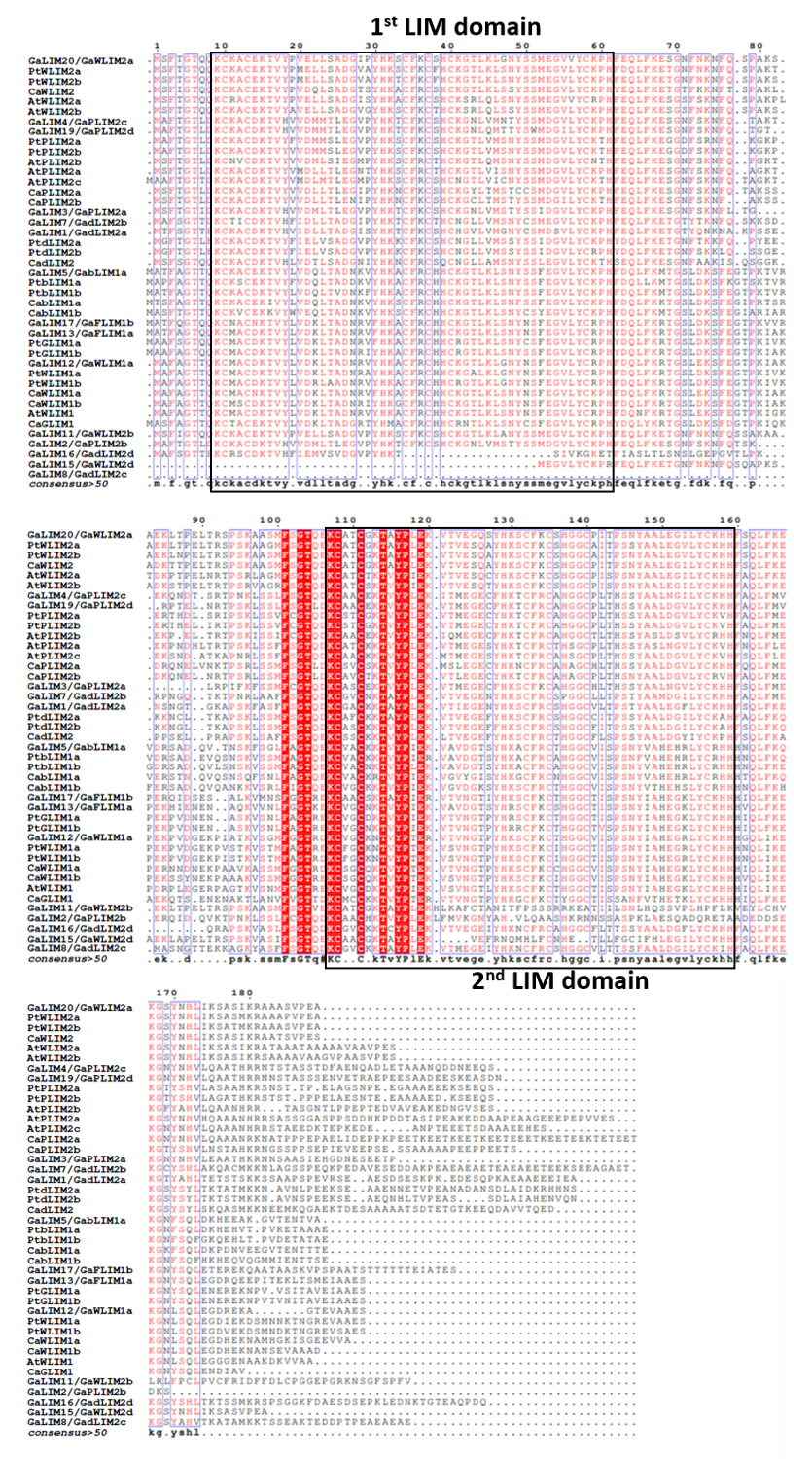


**Supplementary Fig. S3:** Multiple sequence alignment and domain analysis of LIM proteins


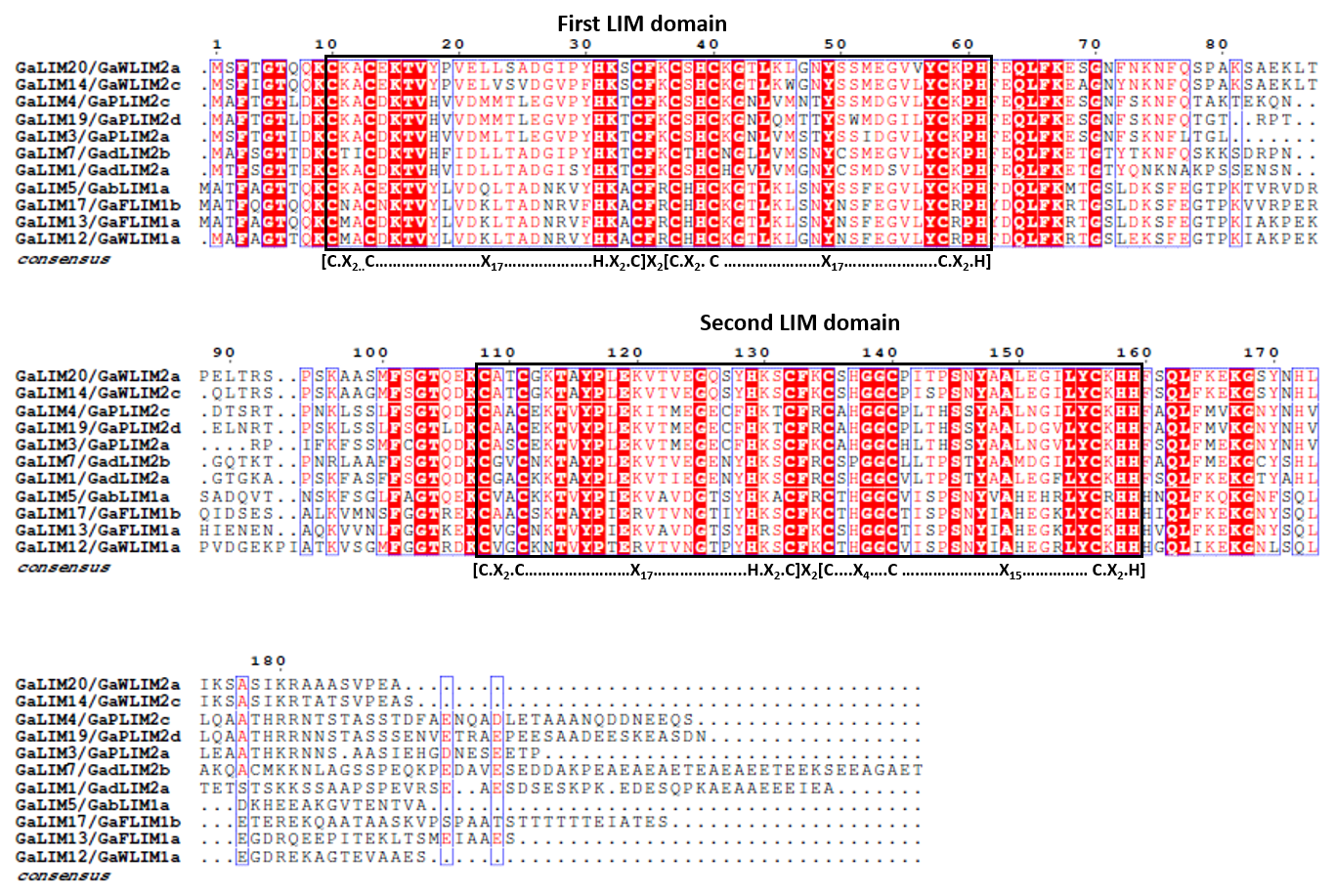


**Supplementary Fig. S4:**Multiple sequence alignment of 2-LIM domain GaLIM proteins


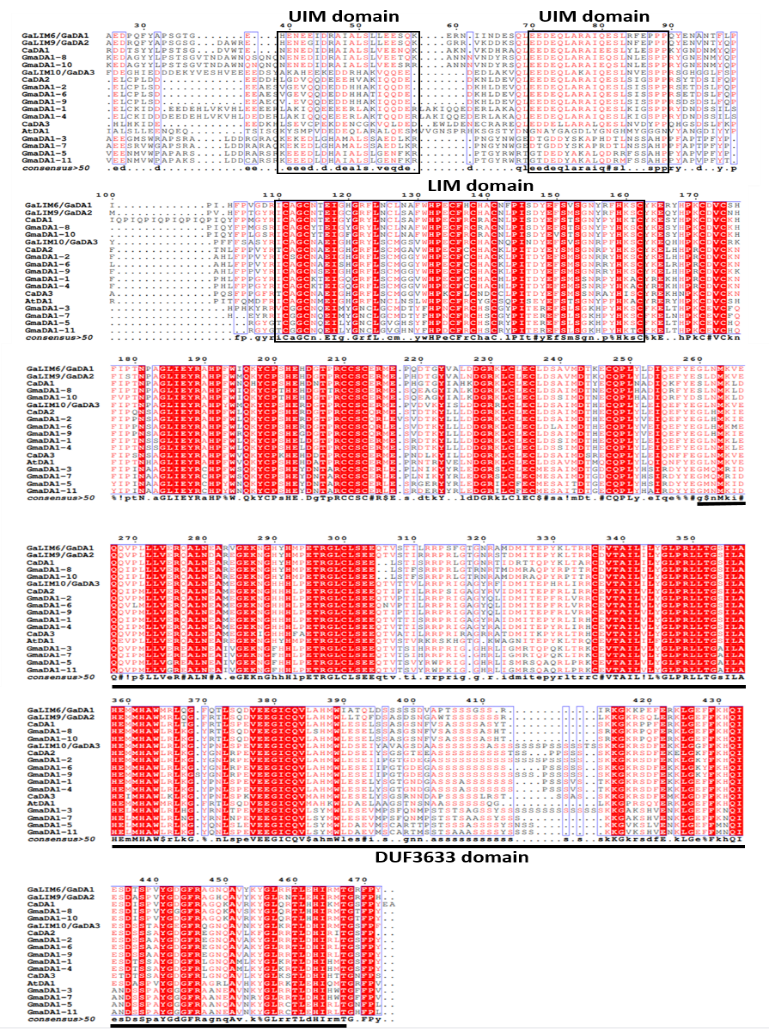


**Supplementary Fig. S5:**Multiple sequence alignment of plant-specific LIM (DA) proteins


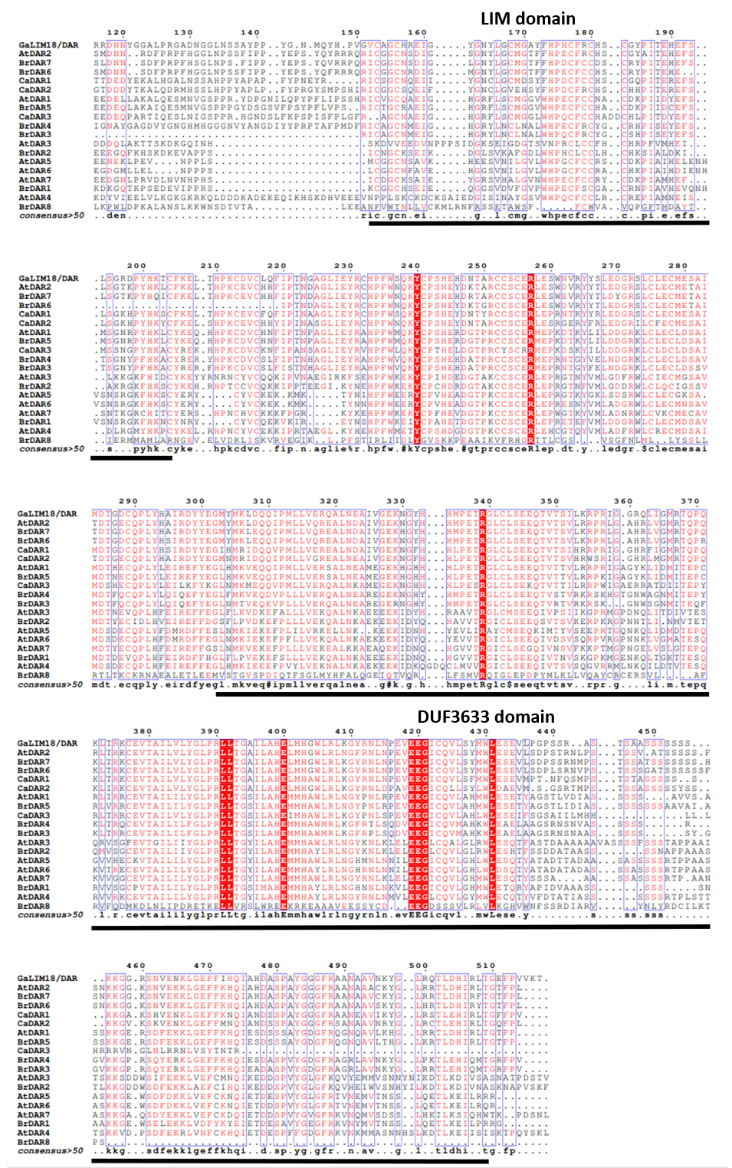


**Supplementary Fig. S6:**Multiple sequence alignment of plant-specific LIM (DAR) proteins

**
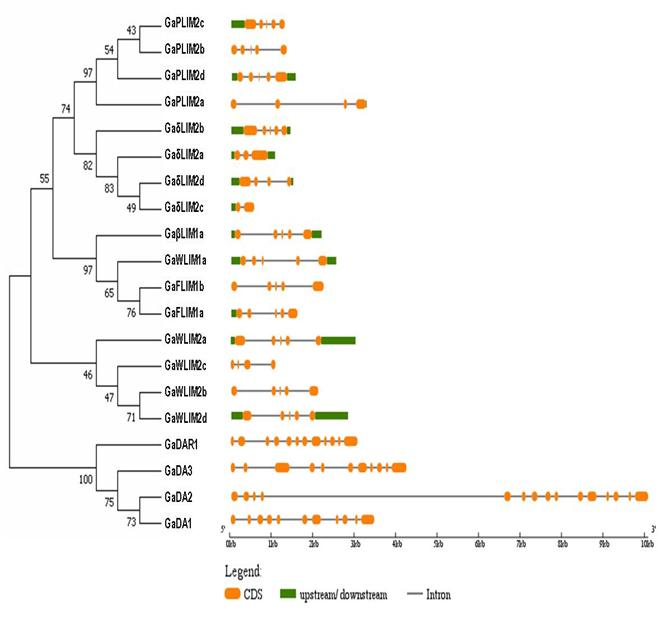
**

**Supplementary Fig. S7:**Genomic organization of LIM genes in *Gossypium arboreum*

**
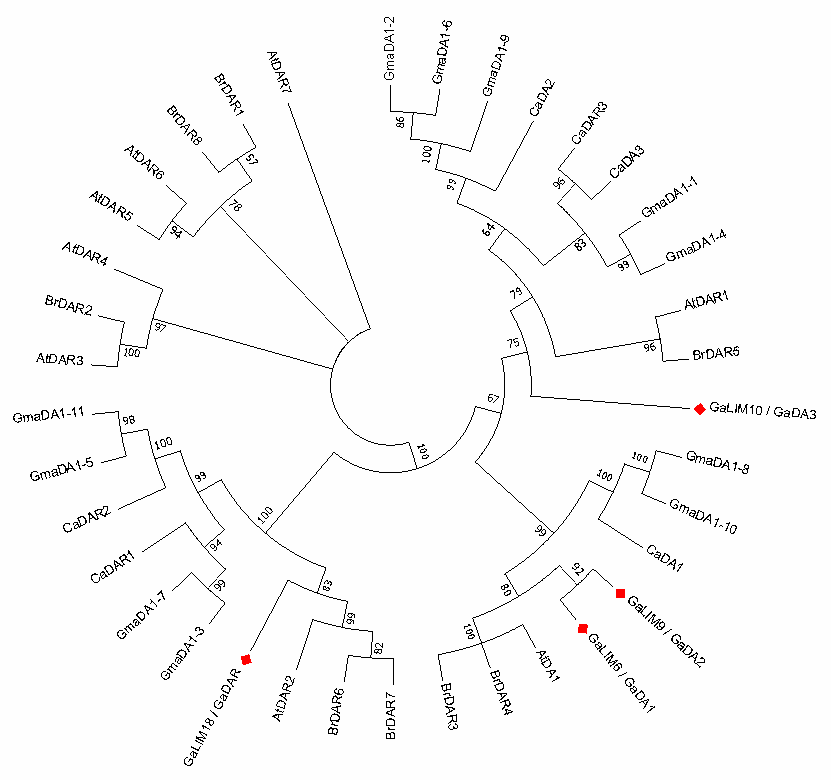
**

**Supplementary Fig. S8:** Maximum likelihood tree representing evolutionary relationship of the identified plant specific *Ga*LIMs compared with that of LIMs from Arabidopsis, Soyabean, Brassica and Chickpea.

**Supplementary Fig.S9** RT-PCR showing organ-specific expression of *LIM* genes in *G. arboreum [100bp ladder was used in the Marker lane with band size gradient of 100, 200, 300, 400, 500 (intense band), 600, 700, 800, 900, 1000 and 1500bp]*


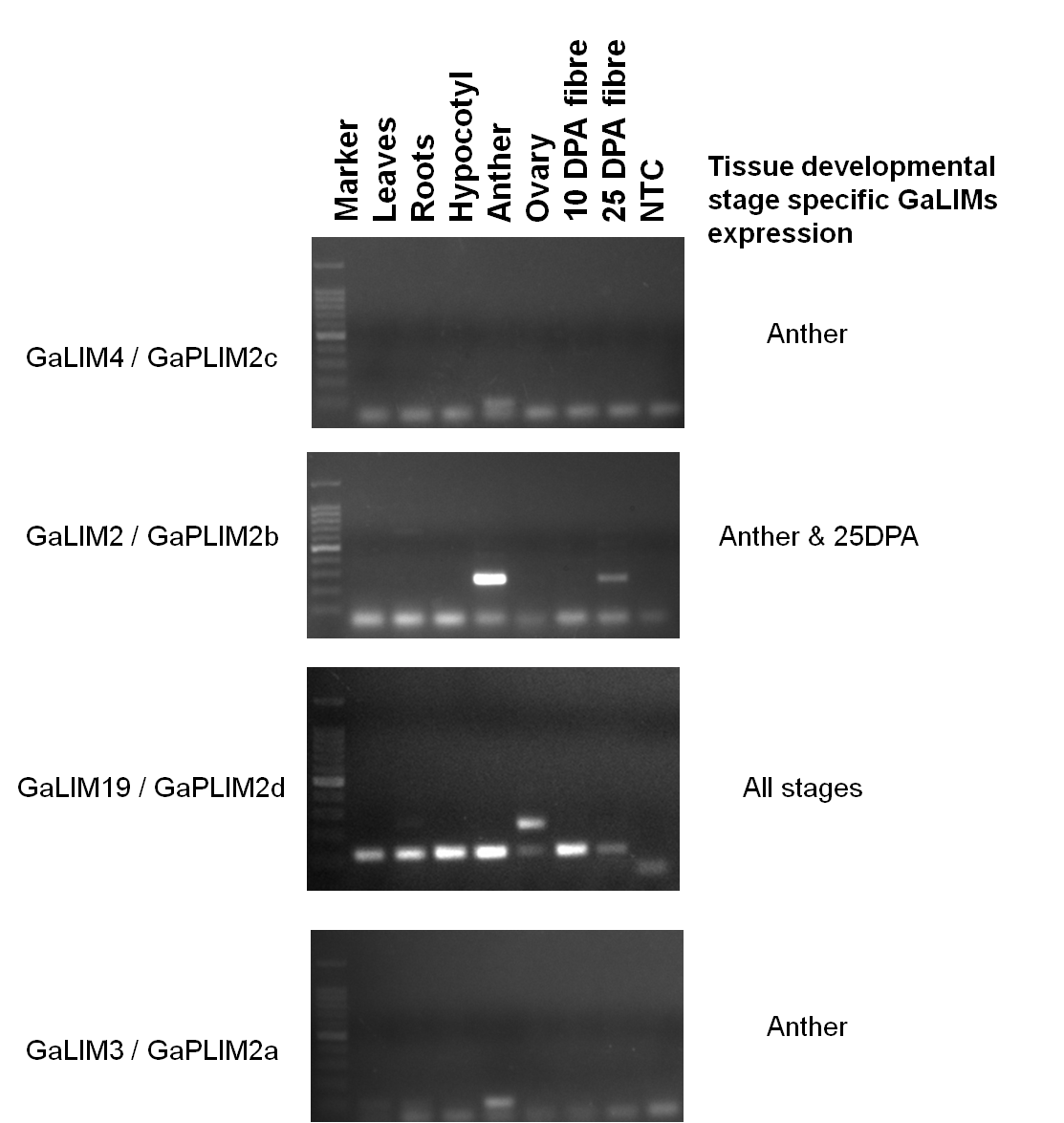


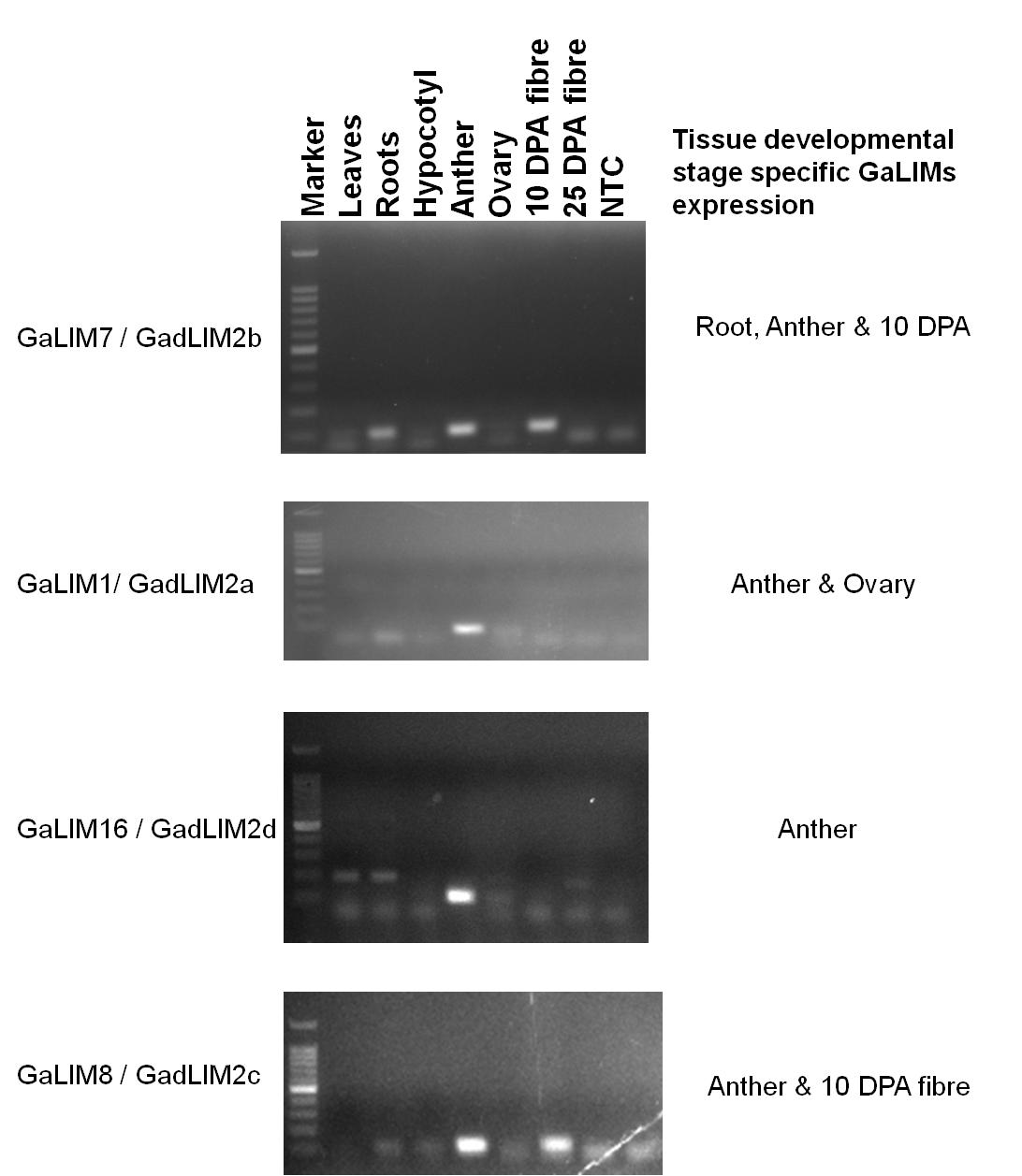


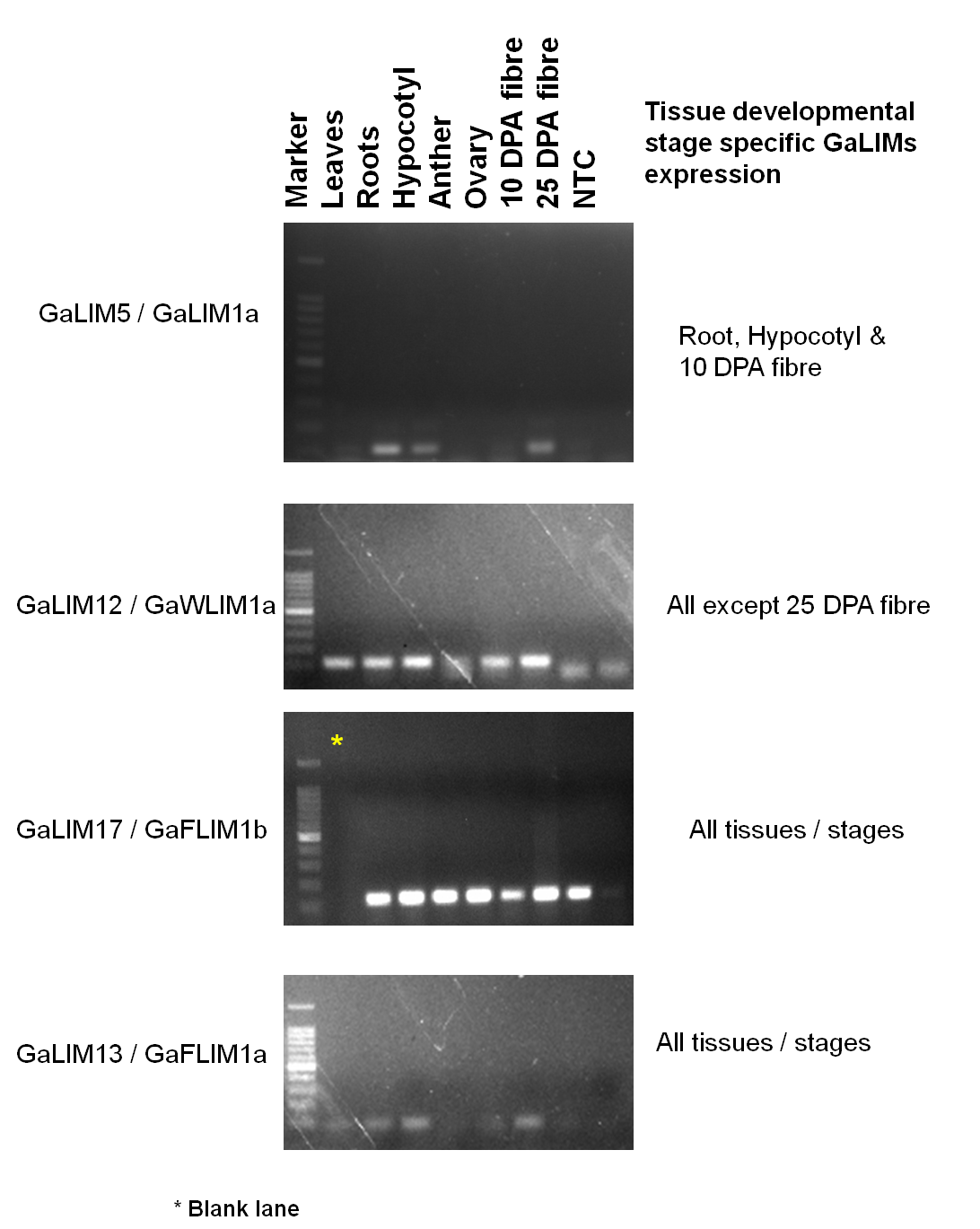


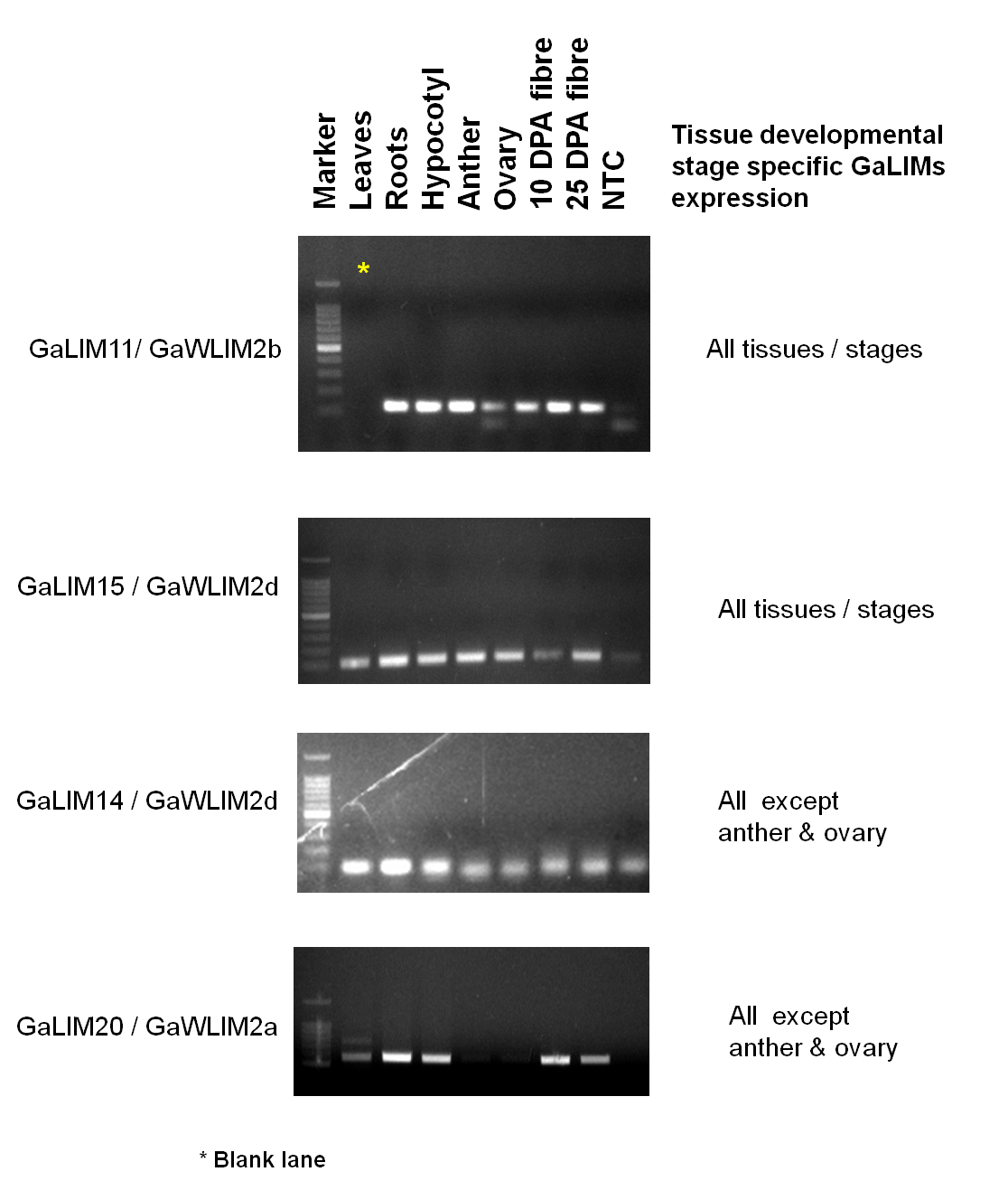


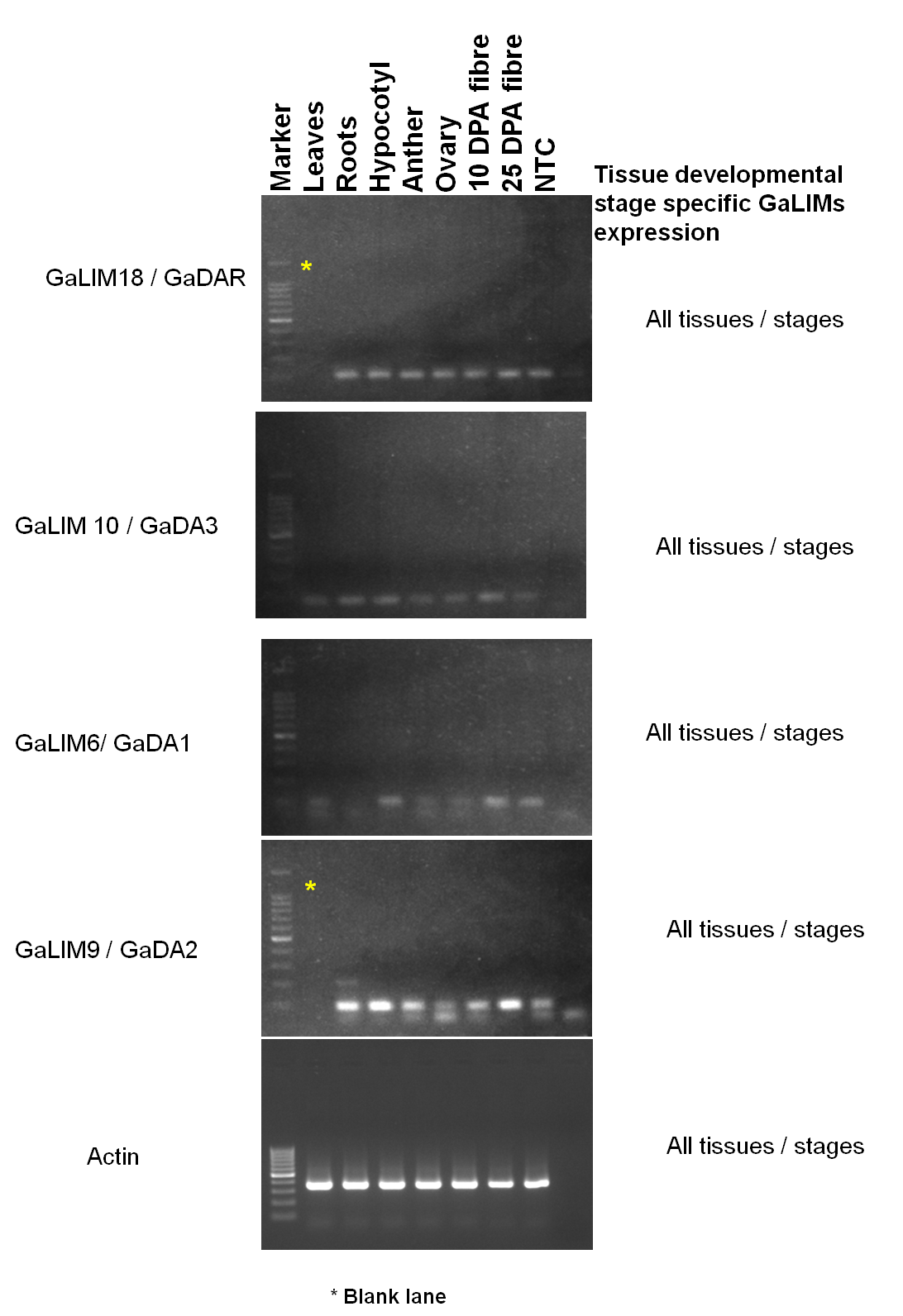

Supplement: Supplementary file 2 — Supplementary Information 2. [file 41598_2021_87934_MOESM2_ESM.docx]
